# Neuronal activity regulates DROSHA via autophagy in spinal muscular atrophy

**DOI:** 10.1038/s41598-018-26347-y

**Published:** 2018-05-21

**Authors:** Inês do Carmo G. Gonçalves, Johanna Brecht, Maximilian P. Thelen, Wiebke A. Rehorst, Miriam Peters, Hyun Ju Lee, Susanne Motameny, Laura Torres-Benito, Darius Ebrahimi-Fakhari, Natalia L. Kononenko, Janine Altmüller, David Vilchez, Mustafa Sahin, Brunhilde Wirth, Min Jeong Kye

**Affiliations:** 1 Institute of Human Genetics, University of Cologne, Cologne, 50931 Germany; 2 Center for Molecular Medicine Cologne, University of Cologne, Cologne, 50931 Germany; 3 Institute for Genetics, University of Cologne, Cologne, 50931 Germany; 4 Cologne Excellence Cluster for Cellular Stress Responses in Aging-Associated Diseases (CECAD), University of Cologne, 50931 Cologne, Germany; 5 Cologne Center for Genomics (CCG), University of Cologne, 50931 Cologne, Germany; 6 Department of Neurology, The F.M. Kirby Center for Neurobiology, Boston Children’s Hospital, Harvard Medical School, Boston, MA 02115 USA; 7 Center for Rare Diseases, University Hospital Cologne, Cologne, Germany

**Keywords:** Macroautophagy, Mechanisms of disease, miRNAs, Neurodegeneration

## Abstract

Dysregulated miRNA expression and mutation of genes involved in miRNA biogenesis have been reported in motor neuron diseases including spinal muscular atrophy (SMA) and amyotrophic lateral sclerosis (ALS). Therefore, identifying molecular mechanisms governing miRNA expression is important to understand these diseases. Here, we report that expression of DROSHA, which is a critical enzyme in the microprocessor complex and essential for miRNA biogenesis, is reduced in motor neurons from an SMA mouse model. We show that DROSHA is degraded by neuronal activity induced autophagy machinery, which is also dysregulated in SMA. Blocking neuronal activity or the autophagy-lysosome pathway restores DROSHA levels in SMA motor neurons. Moreover, reducing DROSHA levels enhances axonal growth. As impaired axonal growth is a well described phenotype of SMA motor neurons, these data suggest that DROSHA reduction by autophagy may mitigate the phenotype of SMA. In summary, these findings suggest that autophagy regulates RNA metabolism and neuronal growth via the DROSHA/miRNA pathway and this pathway is dysregulated in SMA.

## Introduction

microRNAs (miRNAs) are a sub-set of non-coding RNAs, which bind to the 3′-untranslated region of mRNAs, and act as translational repressors. miRNAs play a significant role in a broad range of cellular and developmental processes such as neuronal development^1^, learning and memory^2^ and synaptic plasticity^3^. Dysregulated miRNA expression and mutations of genes involved in miRNA biogenesis are reported in motor neuron disorders such as spinal muscular atrophy (SMA)^4–6^ and amyotrophic lateral sclerosis (ALS)^7–11^. However, little is known about the molecular mechanisms driving miRNA dysregulation in these neurological disorders. Expression of functional miRNAs is tightly regulated in many different steps. Briefly, primary miRNA transcripts are processed by a series of RNases such as DROSHA, DICER1 and AGO2. Only the mature form of miRNAs can form the RNA-induced silencing complex (RISC) and function as a translational repressor^12^.

DROSHA regulates the first step of miRNA biogenesis. It forms a complex with DiGeorge syndrome chromosomal region 8 protein (DGCR8) and processes primary miRNAs (pri-miRNAs) to hairpin-shaped precursor forms (pre-miRNAs). Thus, the majority of miRNAs are processed by the DROSHA/DGCR8 complex (also called the microprocessor complex)^13^. In addition to miRNAs, DROSHA can process other types of RNAs including messenger RNAs (mRNAs) and ribosomal RNAs (rRNAs)^14^. This indicates that proper function of DROSHA is crucial for cells. Indeed, *Drosha* knockout cells show impaired proliferation^15^, and *Dgcr8* null mice are early embryonically lethal (~E6.5)^16^. Moreover, DROSHA controls neurogenesis via processing mRNAs of Neurogenin-2 and Nuclear Factor IB^17,18^. As proper function of DROSHA is important for cellular physiology, *Drosha* expression is tightly regulated via multiple mechanisms including alternative splicing, post-translational modifications and protein degradation pathways^19–23^. Taken together, these findings highlight the importance of DROSHA for development, differentiation and cellular homeostasis.

Spinal muscular atrophy (SMA) is an inherited neuromuscular disorder, characterized by dysfunction/loss of motor neurons and muscle weakness. SMA is caused by mutation/deletion of the *SMN1* (survival motor neurons 1) gene, while disease severity inversely correlates with the number of a mainly non-functional *SMN2* copy gene^24,25^. Despite advanced understanding of the genetics in SMA, no efficient therapy was available for this devastating disease until recently^26–29^. Only lately, splicing correcting antisense oligonucleotide-based therapy has shown promising results in SMA patients and has thus been approved by the FDA and EMA^28,30,31^. Survival motor neuron (SMN), the protein product of *SMN1*, plays various roles including mRNA splicing, snRNP biogenesis and trafficking of polyA-tailed mRNAs to axon terminals^32–34^. Moreover, dysregulated miRNA expression has been reported in SMA animal models and SMN deficient cells including neurons from *C*. *elegans*, neurons and muscles from mice, as well as fibroblasts and serum from patients^4,5,35,36^. However, the molecular mechanisms underlying dysregulated miRNA expression in SMA are poorly defined.

Dysregulated autophagy has been implicated in SMA. Autophagy is a proteolytic physiological process that helps maintain protein homeostasis and energy metabolism via lysosomal degradation^37^. It has been considered that autophagy plays a protective role for neurons^38^. Indeed, mutations in autophagy-related genes are associated with neurological disorders including ALS and frontotemporal dementia^39,40^. While dysregulated autophagy in SMA has been reported, whether it is dysfunctional or hyperactive is not conclusive. Autophagic flux seems obstructed in a motor neuron like NSC-34 cell model of SMA and spinal cords from an SMA mouse model^41^. However, in SMA primary motor neurons obtained from either a mouse model or shRNA-mediated knockdown, the autophagosome formation is enhanced and autophagic flux seems fully functional^42^. Indeed, inhibition of hyperactive autophagy improved motor function and survival in an SMA mouse model^43^. This implies that hyperactive autophagy pathway contributes to SMA pathology. Taken together, current data suggest that the pathophysiology of the autophagy pathway caused by SMN deficiency may be cell type specific and contributes to SMA.

In this study, we aim to understand the molecular mechanism that underlies dysregulated miRNA expression in the motor neuron disease SMA.

## Results

### DROSHA level is reduced in SMA motor neurons

**Figure 1:**
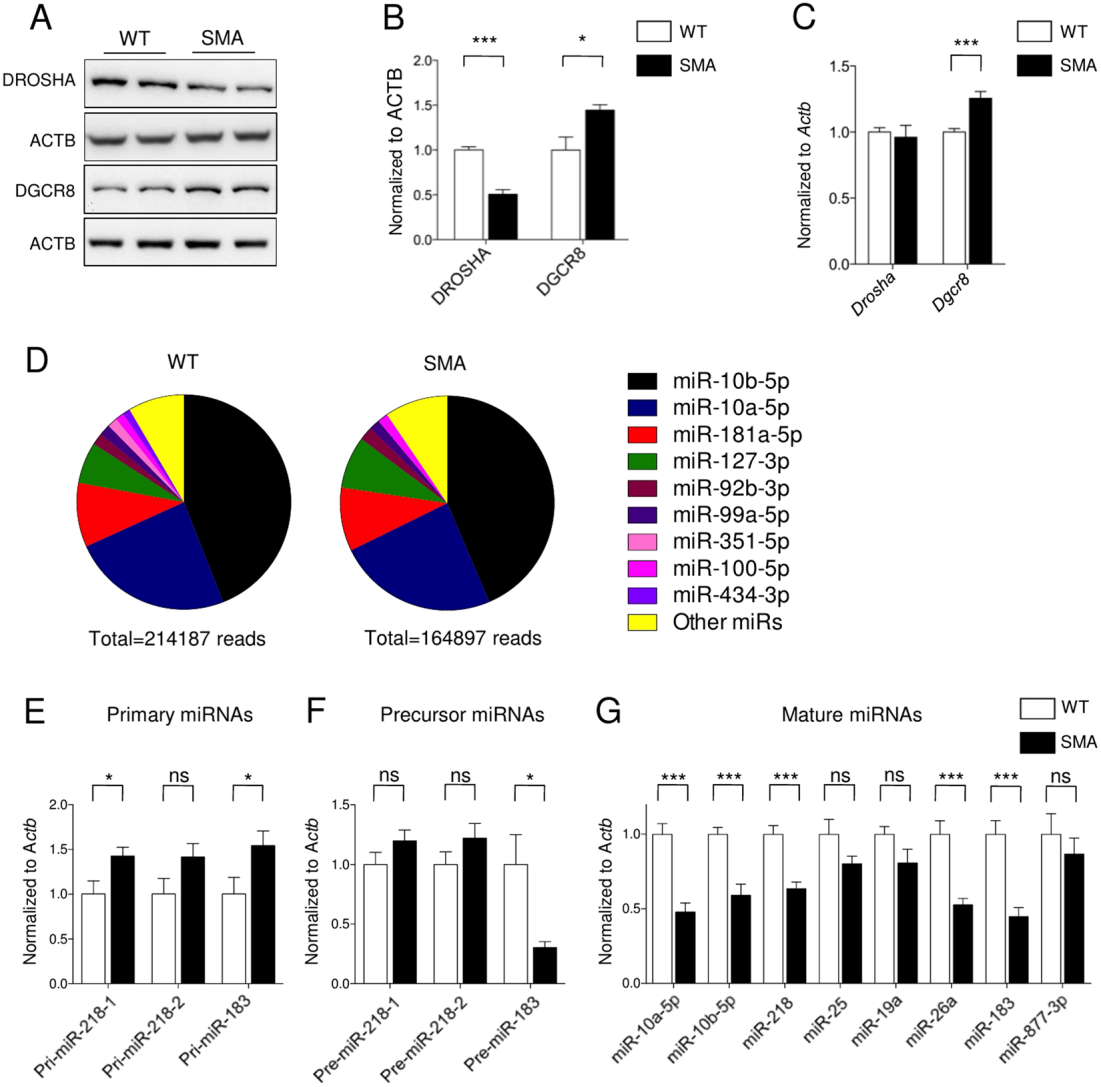
The expression of DROSHA/DGCR8 is dysregulated in SMA motor neurons. (**A**) Western blots of DROSHA, DGCR8 and ACTB in 10DIV motor neurons (**B**) Quantification of Western blots, n = 12 (WT), n = 11 (SMA) for DROSHA, n = 4 (WT and SMA) for DGCR8. Each sample represents an individual embryo. (**C**) mRNA levels of *Drosha* and *Dgcr8* were measured by qRT-PCR in 10DIV motor neurons: n = 20 (WT) and n = 12 (SMA) (**D**) Pie charts represent the composition of miRNAs in 10DIV motor neurons. miRNAs account for less than 1% of total reads were grouped as other miRs. Deep sequencing data show that total number of reads of miRNAs are reduced in SMA. (**E**) Bar graph representing qRT-PCR of primary miRNA transcripts: n = 14 (WT) and n = 13 (SMA) (**F**) Precursor miRNA levels: n = 15 (WT) and n = 18 (SMA) for miR-218-1 and miR-218-2, n = 10 (WT and SMA) for miR-183 (**G**) Mature miRNA levels: n = 34 (WT, except miR-10a-5p, miR-10b-5p and miR-218), n = 22 (SMA, except miR-10a-5p, miR-10b-5p and miR-218), n = 12 (WT, miR-218) and n = 10 (SMA, miR-218), n = 10 (WT and SMA, miR-10a-5p and miR-10b-5p) Data are represented as mean±SEM, Statistical significance is determined with t-test, *p < 0.05 and ***p < 0.001. ns = not significant.

To understand the mechanisms underlying the dysregulated miRNA expression in SMA, we measured the levels of proteins relevant in miRNA homeostasis in spinal motor neurons isolated from E13.5 WT and SMA mice (*Smn*^−/−^;*SMN2*^tg/0^) at 10 days *in vitro* (10DIV) culture (Supplementary Fig. 1). While the protein levels of AGO2, XRN1, ERI1 and DICER1 were unchanged (Supplementary Fig. 2), DROSHA levels were reduced and DGCR8 levels were increased in SMA motor neurons (Fig. 1A,B). DROSHA and DGCR8 work as a complex in the first step of miRNA biogenesis, and they regulate the expression of each other post-transcriptionally. DROSHA cleaves *Dgcr8* mRNA, and DGCR8 stabilizes DROSHA upon binding^44^.

To better understand how these proteins regulate one another in SMA, we first measured mRNA levels of *Drosha* and *Dgcr8* in WT and SMA motor neurons. If SMA primarily results in DROSHA reduction, the amount of *Dgcr8* mRNA and protein product should both be increased. In contrast, if increases in DGCR8 level are the primary change in SMA, DGCR8 would be expected to stabilize DROSHA resulting in increased level of DROSHA. We found that expression of *Dgcr8* mRNA was increased, while *Drosha* mRNA levels were not altered in SMA motor neurons (Fig. 1C). Therefore, these data suggest that reduced DROSHA levels might be the primary event in SMA motor neurons. In addition, we tested whether DROSHA reduction is directly related to SMN levels or phenotype of SMA motor neurons. We knocked down *Smn* with an siRNA, and measured DROSHA levels 72 hours later. DROSHA levels were unaltered in *Smn* knockdown (KD) motor neurons (Supplementary Fig. 3A). In addition, we confirmed that an siRNA treatment reduced SMN levels about ~50% compared to controls, which is similar to SMN levels in SMA motor neurons (~40% compared to WT motor neurons) (Supplementary Fig. 3A,B). This data implies that DROSHA is not directly regulated by SMN, but its dysregulation is rather a phenotype of SMA motor neurons.

### miRNA expression is altered in SMA motor neurons

We further systemically analysed the miRNA expression in 10DIV WT and SMA motor neurons with next generation sequencing. We profiled miRNAs from three independent biological samples (1 µg total RNA per sample). The total number of reads of mature miRNA was reduced by about 23% in SMA motor neurons, suggesting DROSHA dysfunction in SMA (Fig. 1D). Interestingly, the complexity of miRNA expression is rather simple in motor neurons. 251 miRNAs were detected in WT motor neurons, and 9 miRNAs account for more than 92% of miRNAs. Among them, 68% of miRNAs are miR-10a-5p and miR-10b-5p, and 9% are miR-181a-5p. 242 miRNAs showed less than 1% of total reads. In SMA motor neurons, it is even simpler. Among 215 detected miRNAs, only 7 miRNAs are expressed higher than 1% of total miRNA reads (Fig. 1D and Supplementary dataset). Our data is consistent with previous reports showing predominant expression of miR-10a/b in spinal cords of zebrafish, mouse and human^45–47^. We further measured primary, precursor and mature forms of a few known miRNAs using quantitative real time PCR (qRT-PCR). As expected, we found that primary transcript of miRNAs are accumulated, and precursor and mature miRNAs are reduced in SMA motor neurons (Fig. 1E–G). These data support that the DROSHA/DGCR8 complex is dysregulated in SMA motor neurons. In addition, we measured the expression of non-DROSHA processed miRNA (mirtron), miR-877-3p as a control^48^. While the expression level of miR-877-3p was high in motor neurons due to qRT-PCR, its level was not altered in SMA (Fig. 1G).

### *Drosha* knockdown in WT motor neurons partially recapitulates dysregulated miRNA expression in SMA motor neurons

**Figure 2:**
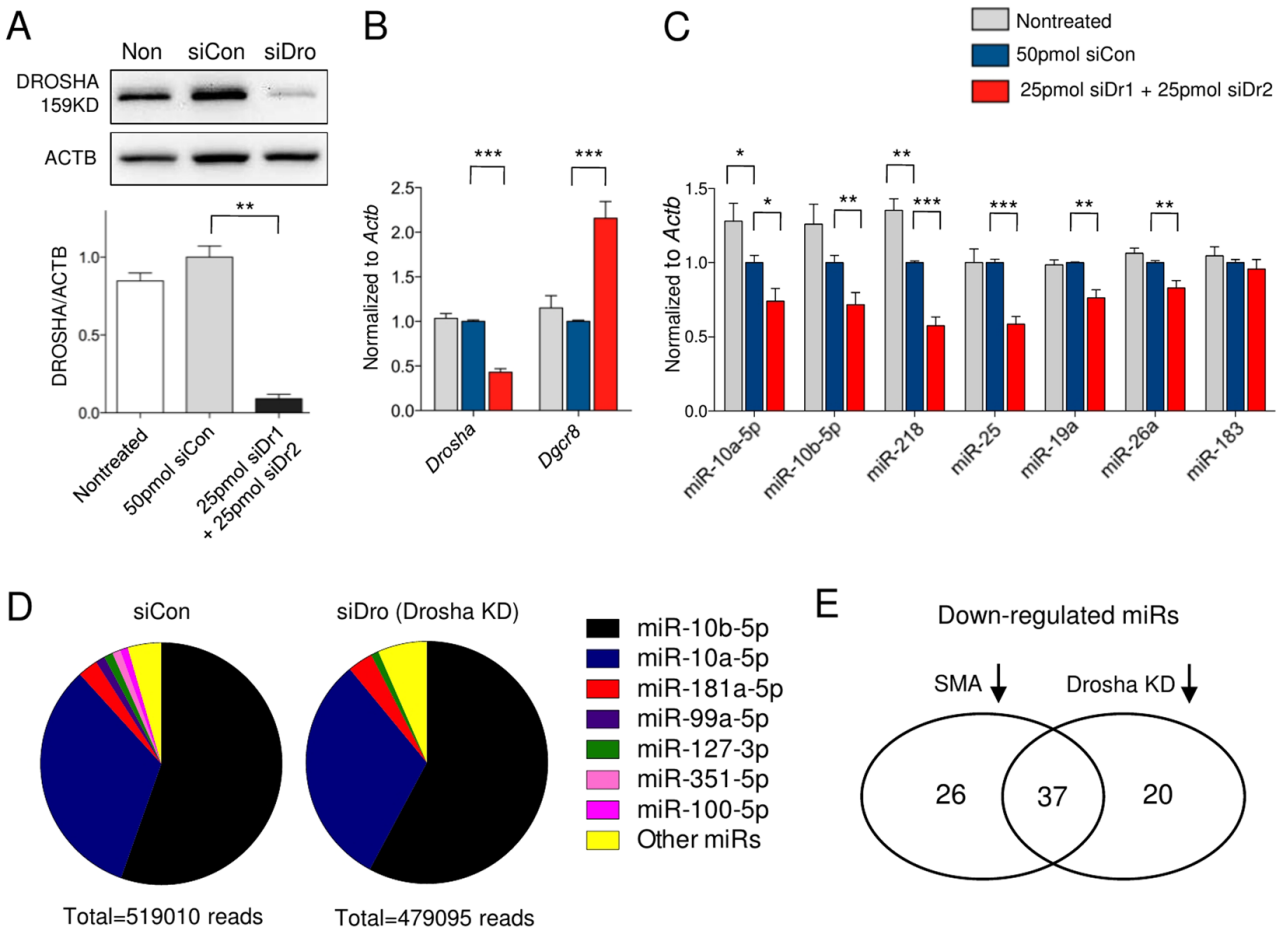
DROSHA reduction contributes to miRNA dysregulation in SMA motor neurons. (**A**) Successful *Drosha* knockdown (KD) was achieved with combining two different *Drosha* siRNAs. siRNAs were transfected to 3DIV motor neurons and KD was confirmed after 72 hours: n = 3 (**B**) mRNAs levels of *Drosha* and *Dgcr8* were measured by qRT-PCR in *Drosha* KD motor neurons: n = 10 (**C**) miRNA levels in 6DIV motor neurons: n = 12 (WT and SMA, except miR-10a-5p and miR-10b-5p), n = 16 (WT and SMA, miR-10a-5p and miR-10b-5p) (**D**) Pie charts represent the composition of miRNAs in 6DIV motor neurons. miRNAs account for less than 1% of total reads were grouped as other miRs. (**E**) Venn diagram shows that 37 common miRNAs were down regulated in SMA and *Drosha* KD neurons compared their controls. Data are represented as mean±SEM, Statistical significance is determined with t-test, *p < 0.05, **p < 0.01 and ***p < 0.001.

To test whether reduced DROSHA levels contribute to dysregulated miRNA expression in SMA motor neurons, we knocked down *Drosha* with siRNAs in WT motor neurons (Fig. 2A). We first optimized siRNA-mediated KD against *Drosha* in cortical neurons, and found that a combination of two siRNAs against *Drosha* show robust KD efficiency (Supplementary Fig. 4). *Drosha* KD elevated *Dgcr8* mRNA expression in WT motor neurons (Fig. 2B), similar to what we observed in SMA motor neurons (Fig. 1C). In addition, *Drosha* KD also partially recapitulated dysregulated miRNA expression observed in SMA motor neurons (Fig. 2C). miR-10a-5p, mir-10b-5p, miR-26a and miR-218 levels were reduced in both *Drosha* KD and SMA motor neurons, while other miRNAs such as miR-25, miR-183 and miR-19a were not altered in the same way by *Drosha* KD as we have observed in SMA motor neurons (Fig. 2C). Furthermore, we also systemically sequenced miRNAs in 6DIV control and *Drosha* KD motor neurons. Compared to 10DIV motor neurons, the portion of miR-10a/b was even higher, and the profile of miRNA expression was simpler (Fig. 2D and Supplementary dataset). Although we did not observe clear reduction in total number of miRNAs after 3 days of DROSHA reduction, we confirmed that a large portions of miRNAs were reduced in both SMA and *Drosha* KD motor neurons compared to their controls (Fig. 2D,E). In 10DIV motor neurons, 279 miRNAs were detected, of which 63 miRNAs were reduced and 9 miRNAs were increased in SMA samples with more than 10 reads. In 6DIV motor neurons, 309 miRNAs were detected, of which 57 miRNAs were decreased and 7 miRNAs were elevated in *Drosha* KD. Among decreased miRNAs, 37 miRNAs were common (Fig. 2E and Supplementary dataset). However, among elevated miRNAs, none were common between the two sets of samples. Together, these data suggest that reduction in DROSHA partially contributes to dysregulated miRNA expression in SMA motor neurons but that other mechanisms are also likely involved in miRNA expression changes in SMA.

Previously, we have reported that various SMN-deficient cells including shRNA-mediated *Smn* KD neurons and fibroblasts from SMA patients showed elevated miR-183 expression^4^. To understand the discrepancies in our work, we measured the levels of miRNAs in miR-183 cluster in muscle cells from the SMA mice and SMN depleted muscle cell lines. In agreement with the previous results, the miR-183 expression was increased in SMA muscle cells, while DROSHA amount was not altered (Supplementary Fig. 5). These findings suggest that there is a motor neuron-specific mechanism for regulation of miRNA expression.

### Neuronal activity reduces DROSHA levels in motor neurons

**Figure 3:**
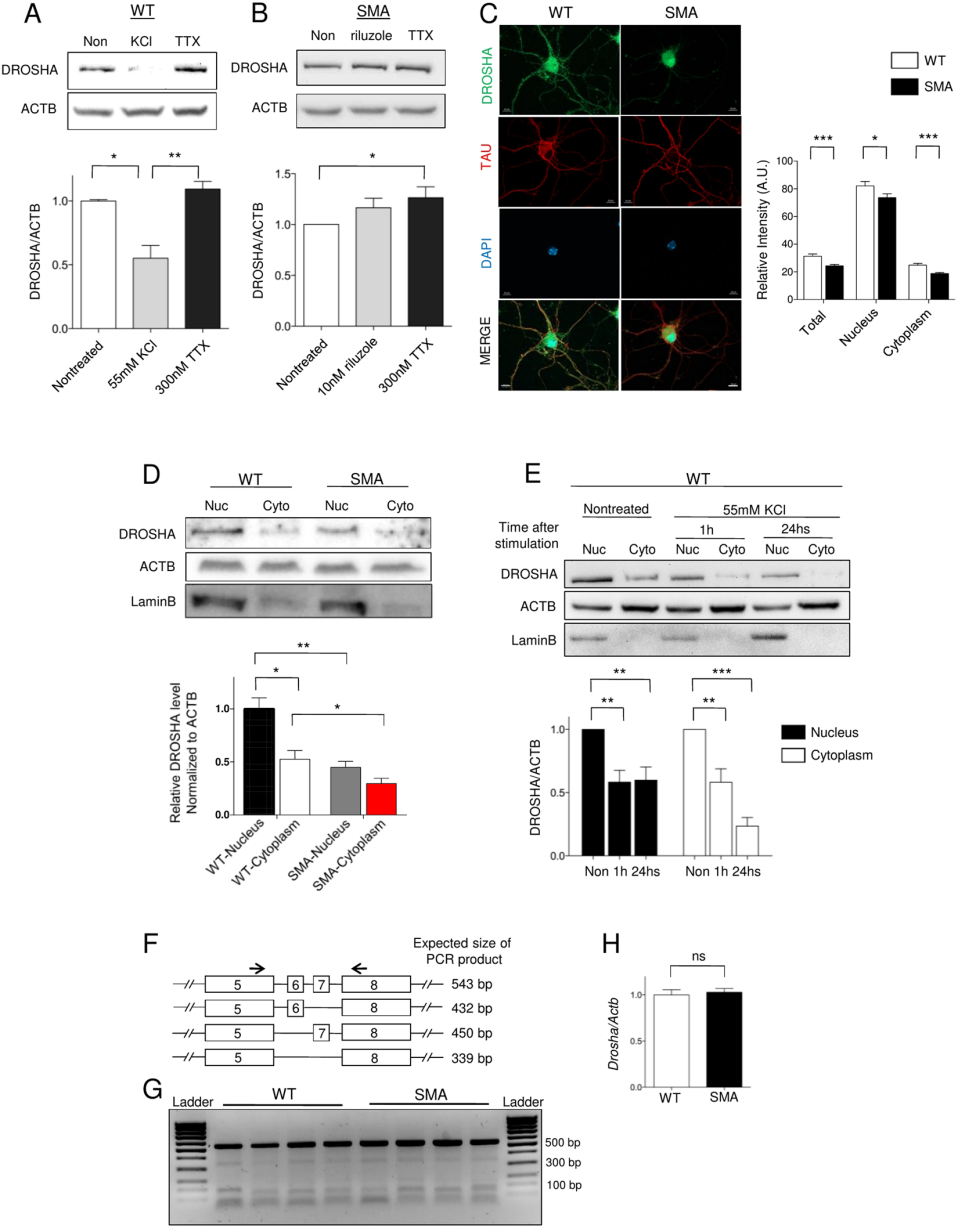
Neuronal activity decreases DROSHA levels in motor neurons. (**A**) Western blot analysis of DROSHA levels in 10DIV WT motor neurons treated with 55 mM KCl or 300 nM tetrodotoxin (TTX) for 24 hours: n = 5. (**B**) Western blot analysis of DROSHA levels in 10DIV SMA motor neurons treated with 10 nM riluzole or 300 nM TTX for 96 hours: n = 9 (**C**) Images show that DROSHA is detected in nucleus and cytoplasm of motor neurons. Cytoplasmic DROSHA intensity is reduced in SMA: n = 113 (WT) and n = 98 (SMA) from 9 (WT) and 8 (SMA) biological samples (**D**) Western blot analysis and quantification of DROSHA levels in nucleus and cytoplasm of WT and SMA motor neurons: n = 7 (WT) and n = 6 (SMA) (**E**) Western blot analysis and quantification of DROSHA levels in nucleus and cytoplasm of depolarized neurons. Neurons were depolarized with 55 mM KCl. LaminB and ACTB confirm that subcellular fractionation was successful: n = 5 (**F**) Primers to amplify exon 7 related splicing variants (**G**) PCR shows that exons 5/6/7/8 containing splicing variant was the major form in motor neurons and SMN deficiency does not alter the exon 7-related splicing. PCR was performed 39 cycles and PCR product was confirmed with Sanger sequencing: n = 4 (**H**) qRT-PCR confirms that the amount of transcripts containing exon 7 did not alter in SMA: n = 4, Data are represented as mean±SEM. Statistical significance is determined with t-test, *p < 0.05, **p < 0.01 and ***p < 0.001. ns = not significant.

Next, we investigated the molecular mechanisms underlying reduced DROSHA levels in SMA motor neurons. We have previously reported that N-methyl-D-aspartate (NMDA)-mediated neuronal activity suppresses *Drosha* expression in hippocampal neurons^49^. Moreover, SMA neurons display hyperexcitability, and reducing neuronal activity by riluzole ameliorates SMA phenotypes via opening of Ca^2+^-activated small conductance potassium (SK) channels^50,51^. Therefore, we first tested whether DROSHA is also regulated by neuronal activity in WT motor neurons. Indeed, KCl-induced neuronal depolarization reduced DROSHA levels after 24 hours of stimulation, while blocking neuronal activity with tetrodotoxin (TTX) did not change DROSHA levels (Fig. 3A). Next, we pharmacologically reduced or blocked neuronal activity with riluzole or TTX respectively in SMA motor neurons. While riluzole did not increase DROSHA substantially, TTX significantly increased DROSHA in SMA motor neurons (Fig. 3B). From these data, we conclude that neuronal activity decreases DROSHA levels in motor neurons.

### DROSHA is detected in the cytoplasm of motor neurons

DROSHA has long been considered to be a nuclear protein containing a nuclear localization signal in its N-terminal^20^. However, recent reports show that subcellular localization of DROSHA varies among cell types and *Drosha* expression can be regulated by alternative splicing or post-translational modification^19,20,23,52^. Therefore, we checked the subcellular localization of DROSHA in WT and SMA motor neurons. In WT motor neurons, DROSHA can be detected in both the nucleus and cytoplasm including in neurites, while DROSHA in SMA neurites appears to be lower than in WT neurites (Fig. 3C). To confirm our finding with an independent method and quantify the level of reduction, we generated subcellular fractions from WT and SMA motor neurons and measured DROSHA levels. Indeed, we detected DROSHA in cytoplasmic fractions in both WT and SMA motor neurons (~50% compared to nucleus), but levels were reduced in both nuclear and cytoplasmic fractions in SMA motor neurons (Fig. 3D). Successful fractionation was confirmed with the nuclear marker LaminB (Fig. 3D).

### Neuronal activity regulates DROSHA expression in nucleus and cytoplasm of motor neurons

Next, we checked whether the level of DROSHA in different subcellular compartments can be regulated distinctly. We measured DROSHA levels after various durations of depolarization in subcellular fractions. In unstimulated WT motor neurons, DROSHA levels were higher in nuclear fractions (Fig. 3E). One hour after depolarization, DROSHA levels were reduced in both nucleus and cytoplasm to ~60% compared to unstimulated controls. Twenty-four hours after depolarization, cytoplasmic DROSHA levels were further decreased to 20%, while nuclear DROSHA levels were not further decreased (Fig. 3E). As both nuclear and cytoplasmic DROSHA levels were reduced rather quickly after neuronal depolarization, it seems that DROSHA is regulated by neuronal activity independent of their subcellular localization. However, as proteins can traffic in and out of the nucleus, we cannot exclude that DROSHA can be regulated in different subcellular compartments by distinct mechanisms.

### SMN deficiency does not influence Drosha splicing

Recently, various splicing variants of *Drosha* have been reported^19,23^. These splicing variants do not show clear functional differences in miRNA processing, but their subcellular localizations are different. Interestingly, this process seems cell type specific, and the presence of exon7 in *Drosha* mRNA is important for cytoplasmic localization^19,23^. As SMN plays an important role in splicing and *Drosha* splicing variants in motor neurons have not been reported, we investigated the alternative splicing pattern of *Drosha* exon7 in WT and SMA motor neurons. We adopted and modified published primer pairs to examine *Drosha* splicing (Fig. 3F)^19^. We tested whether exon7-related splicing variants exist in motor neurons and whether they are altered in SMA. We found that the major form of *Drosha* mRNA in motor neurons is full-length and that SMN deficiency does not alter exon7-related splicing patterns (Fig. 3G). We also compared the amount of *Drosha* mRNA between WT and SMA motor neurons using qRT-PCR with gene specific primers detecting exon7 and found no difference (Fig. 3H). Therefore, we concluded that SMN-mediated splicing does not influence alternative splicing of *Drosha* exon7 and subcellular localization in motor neurons.

### DROSHA is less stable in SMA motor neurons

**Figure 4:**
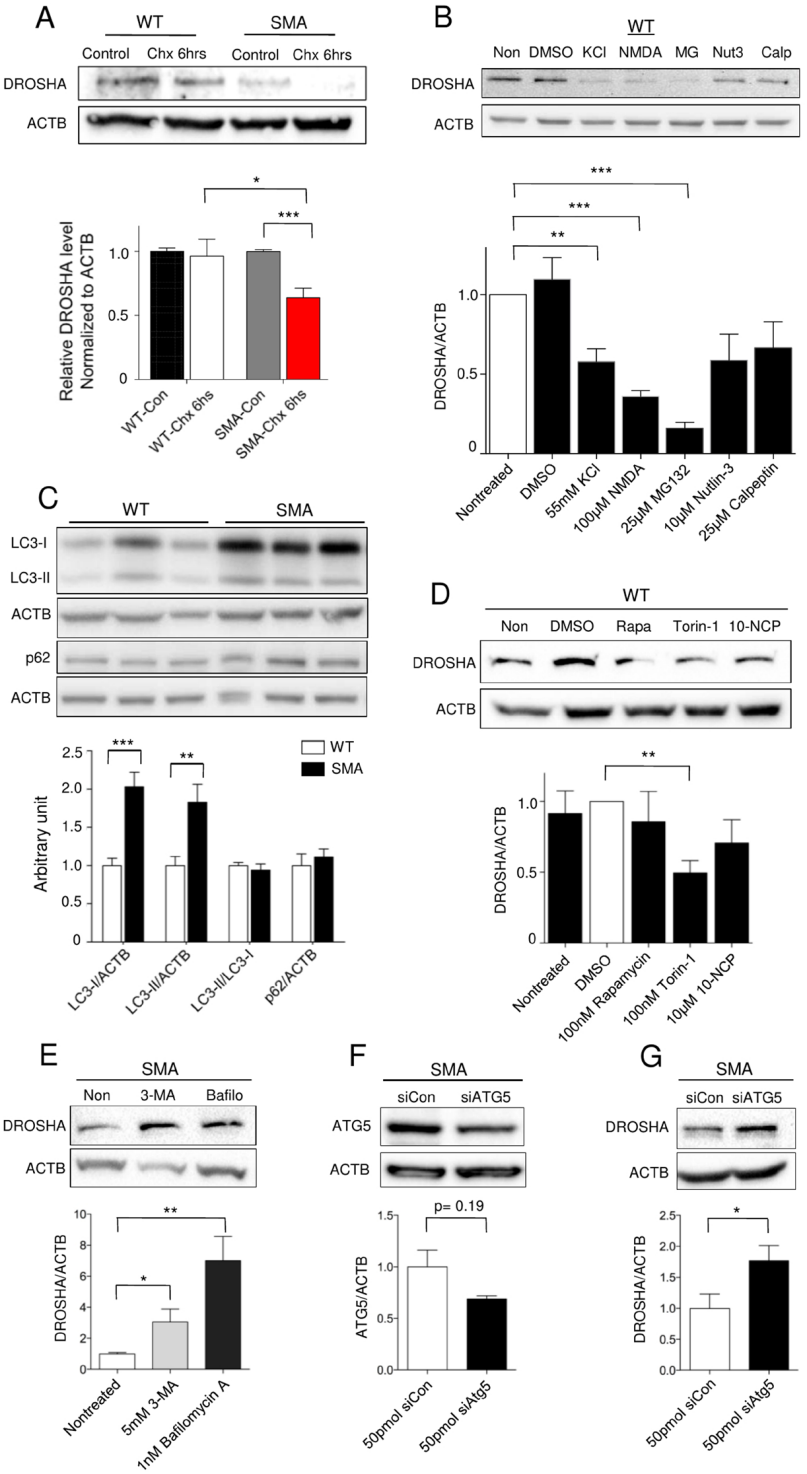
DROSHA is degraded by autophagy in motor neurons. (**A**) Stability of DROSHA protein in 10DIV WT and SMA motor neurons. 20 μg/ml of cycloheximide was treated for 6 hours: n = 8 (WT) and n = 10 (SMA) (**B**) Western blot analysis of DROSHA levels in 10DIV WT motor neurons treated with 25 μM MG132, 10 μM nutlin-3, 25 μM calpeptin, 55 mM KCl or 100 μM NMDA for 24 hours: n = 5. (**C**) Western blot analysis and quantification of LC3-I, LC3-II and p62 levels in 10DIV motor neurons. (**D**) Western blot analysis and quantification of DROSHA levels in 10DIV WT motor neurons treated with autophagy inducers, 100 nM rapamycin, 100 nM Torin-1, and 10 μM 10-NCP for 24 hours: n = 6. (**E**) Western blot analysis and quantification of DROSHA levels in 10DIV SMA motor neurons treated with blockers of autophagic flux, 5 mM 3-methyladenine (3-MA) or 1 nM bafilomycin A for 24 hours: n = 8 (3-MA treated) and n = 9 (non-treated and bafilomycin A treated). (**F**) Western blot analysis shows *Atg5* knockdown (KD) efficiency in SMA motor neurons. 50 pmol of siRNAs (*Atg5* or negative control) were transfected and KD efficiency was confirmed in 48 hours: n = 3. (**G**) *Atg5* KD elevated DROSHA levels in SMA motor neurons after 72 hours of transfection: n = 4. Data are represented as mean±SEM. Statistical significance is determined with t-test, *p < 0.05, **p < 0.01 and ***p < 0.001.

As only DROSHA protein levels are reduced while mRNAs remain unaltered in SMA (Fig. 1A,B), we hypothesized that DROSHA is regulated post-translationally. Therefore, we measured the stability of DROSHA in SMA motor neurons. We blocked protein synthesis with cycloheximide and measured the amount of DROSHA in 10DIV WT and SMA motor neurons. After 6 hours of 20 µg/ml cycloheximide treatment, the amount of DROSHA was clearly reduced in SMA, but not in WT motor neurons (Fig. 4A). These data suggest that the stability of DROSHA is reduced in SMA motor neurons, and that the protein degradation pathway may contribute to the reduced DROSHA amounts.

Next, we investigated the mechanisms by which neuronal activity regulates DROSHA stability. Since we depolarized motor neurons with KCl, which is an unspecific stimulus, we tested whether activation of NMDA receptors also reduce DROSHA. Indeed, NMDA receptor-mediated neuronal activation reduced DROSHA (Fig. 4B). A few mechanisms regulating DROSHA stability have been reported in various cellular contexts. First, DROSHA has been shown to be ubiquitinated by mouse double minute 2 homolog (MDM2, E3 ubiquitin ligase) in response to glucose deprivation with subsequent degradation by the proteasome system^22^. Second, cellular stressors such as heat shock and oxidative stress can decrease DROSHA levels via the p38-MAPK-calpain pathway^21^. Therefore, we tested whether these mechanisms also regulate DROSHA in motor neurons. We treated WT motor neurons with the proteasome inhibitor MG132 (25 μM), the MDM2 inhibitor nutlin-3 (10 μM)^53^, and the calpain inhibitor calpeptin (25 μM)^54^ for 24 hours. Surprisingly, while nutlin-3 and calpeptin did not change the amount of DROSHA significantly, MG132 reduced DROSHA levels after 24 hours of treatment (Fig. 4B and Supplementary Fig. 6). From these data, we conclude that neither MDM2 nor calpain nor the proteasome system do directly regulate DROSHA in motor neurons.

### Autophagy degrades DROSHA in motor neurons

We further searched for the molecular mechanism underlying reduced DROSHA stability in SMA. First, we questioned how inhibiting the proteasome with MG132 can reduce DROSHA levels (Fig. 4B and Supplementary Fig. 6). It has been reported that the proteasome and autophagy pathways interact indirectly with each other and that inhibiting the proteasome pathway with MG132 can enhance autophagy^55,56^. In addition, enhanced autophagosome formation has been reported in SMA motor neurons, and inhibition of hyperactive autophagy delayed motor neuron degeneration and prolonged lifespan of a mild SMA mouse model^42,43^. Based on these findings, we hypothesized that autophagy might regulate DROSHA in SMA motor neurons. To test our hypothesis, we first tested and confirmed that LC3-II, an indicator of autophagy activity and a marker of autophagosomes, is elevated in SMA motor neurons (Fig. 4C). Next, as autophagy plays an important role for clearance of proteins, we tested whether autophagy regulates DROSHA level. We pharmacologically induced autophagy with rapamycin, Torin-1, and 10-(4′-(N-diethylamino) butyl)-2-chlorophenoxazine hydrochloride (10-NCP) in WT motor neurons. Indeed, 100 nM Torin-1 treatment for 24 hours reduced DROSHA level to less than 50% of vehicle (DMSO)-treated motor neurons (Fig. 4D). However, we did not detect robust reduction of DROSHA level neither with 100 nM rapamycin nor with 10 μM 10-NCP. This could be due to the fact that 100 nM rapamycin and 10 µM 10-NCP were not optimal concentrations to induce autophagy in motor neurons. Therefore, we further increased concentrations of rapamycin and 10-NCP. Interestingly, while higher concentrations of 10-NCP successfully reduced DROSHA levels dose-dependently, higher concentrations of rapamycin still did not reduce DROSHA levels (Supplementary Fig. 7). Next, we pharmacologically blocked autophagic flux in SMA motor neurons using the autophagy inhibitor 3-methyladenine (3-MA) or lysosomotropic agent bafilomycin A. Inhibition of autophagic flux with either inhibitor significantly increased DROSHA levels (Fig. 4E). In addition, using siRNA, we knocked-down *Atg5*, which is necessary for autophagosome formation in SMA motor neurons (Fig. 4F). Although siRNA-mediated KD was not very efficient, we observed elevated DROSHA levels upon *Atg5* KD in SMA motor neurons (Fig. 4G). Based on these findings, we conclude that autophagy degrades DROSHA and that hyperactive autophagy contributes to reduced DROSHA level in SMA motor neurons.

### Neuronal activity induces autophagy to decrease DROSHA expression

**Figure 5:**
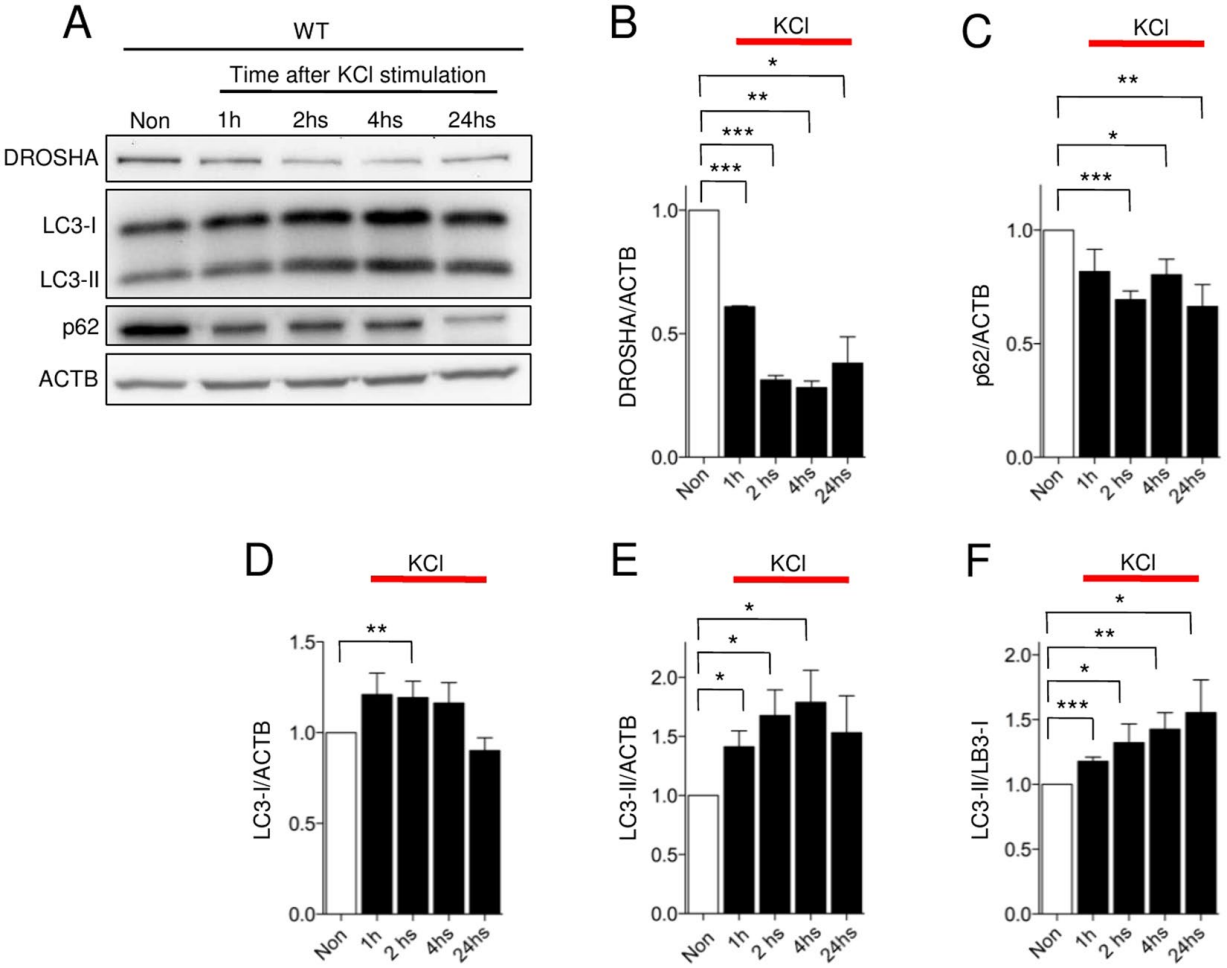
Neuronal activity regulates autophagy and DROSHA. (**A**) Western blot analysis of DROSHA, LC3-I, LC3-II, p62 levels in 10DIV WT motor neurons after 55 mM KCl induced depolarization at different time points. (**B**) Quantification of DROSHA: n = 3 (**C**) Quantification of p62: n = 6, (**D**–**F**) Quantification of LC3-I and LC3-II, and ratio between LC3-II and LC3-I: n = 6. Data are represented as mean ± SEM. Statistical significance is determined with t-test. *p < 0.05, **p < 0.01 and *** p < 0.001.

Next, we investigated whether neuronal activity regulates DROSHA levels via autophagy. It has been reported that neuronal activity induces autophagy via NMDA receptors in hippocampal neurons^57^ and that SMA motor neurons show both abnormal neuronal excitability and autophagy^41,50^. To identify their connections in motor neurons, we depolarized 10DIV WT motor neurons with KCl and measured autophagy and DROSHA levels at different time points (Fig. 5A–F). DROSHA levels decreased quickly after depolarization. We observed the reduction of DROSHA at 1 hour after depolarization, and this lasted up to 24 hours (Fig. 5A,B). Autophagy was similarly increased at 1 hour after depolarization, and this also lasted more than 24 hours (Fig. 5A,D–F). We found that DROSHA levels and autophagy activity were negatively correlated. Moreover, DROSHA levels showed a stronger inverse relationship with LC3-II/I ratio than the well-known autophagy substrate p62/SQSTM1 (Fig. 5A,C). This implies that DROSHA is a strong target of autophagy. Taken together, these data suggest that neuronal activity increases autophagy activity to degrade DROSHA in motor neurons.

### DROSHA is co-localized with endosome-autophagosome markers upon neuronal activation

**Figure 6:**
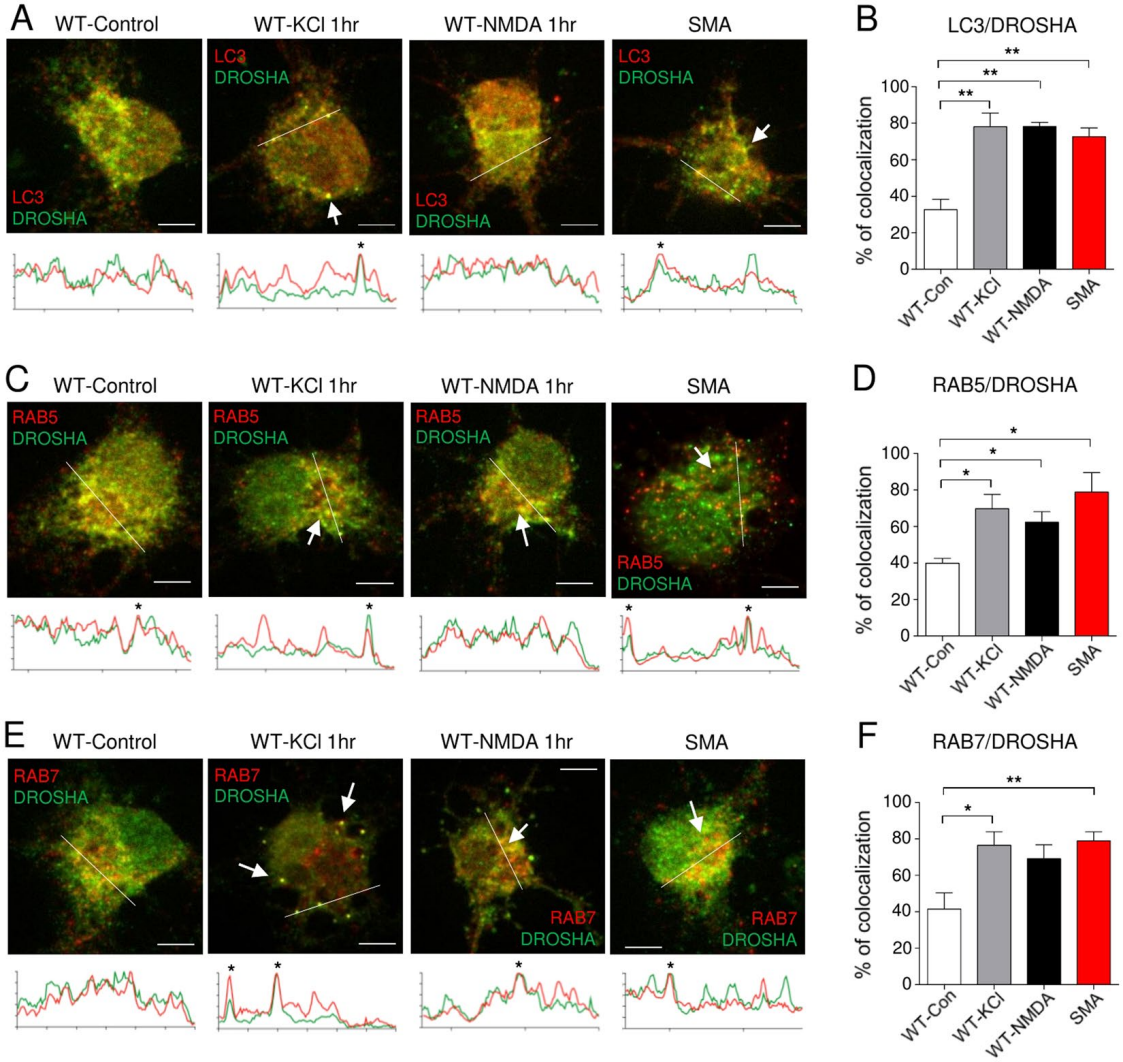
DROSHA is localized in endosomal-autophagy compartments in stimulated and SMA motor neurons. Subcellular localization of DROSHA was checked with vesicular markers. WT neurons were stimulated with 55 mM KCl or 100μM NMDA for 1 hour. SMA neurons were not stimulated. (**A**,**C**,**E**) Images show co-localization of DROSHA with vesicular markers. Arrows point overlapped signals. green: DROSHA, red: vesicular organelle markers, listed in the figure. *Overlapped signals. (**B**,**D**,**F**) Percentage of neurons showing co-localization of DROSHA with vesicular markers. Neurons are individually analyzed. At least 3 independent biological samples were analyzed and 136 to 275 neurons were analyzed per group. Data are represented as mean ± SEM. Statistical significance is determined with t-test. *p < 0.05 and **p < 0.01.

To investigate how autophagy regulates cytoplasmic DROSHA in motor neurons in response to neuronal activity, we co-stained DROSHA with various vesicular organelle markers including LC3 (autophagosomes and amphisomes), p62/SQSTM1 (substrate of autophagy, detected in autophagosomes and amphisomes), RAB5 (recycling endosomes), RAB7 (late endosome and amphisome), EEA1 (early endosomes) and LAMP1 (lysosomes). In response to neuronal activation, DROSHA co-localized with LC3, RAB5 and RAB7 (Fig. 6A–F and Supplementary Figs 8–11). These data suggest that neuronal stimulation relocates DROSHA to vesicular compartments of the autophagy-endosomal pathway. We next checked localization of DROSHA in SMA motor neurons. We observed that DROSHA is highly co-localized in vesicular compartments of the autophagy-endosomal pathway in SMA, comparable to stimulated WT motor neurons (Fig. 6A–F and Supplementary Figs 8–11). In particular, upon depolarization, DROSHA is co-localized at the highest levels with RAB7, a marker of late endosomes and amphisomes (Fig. 6E,F, and Supplementary Fig. 10). These findings support our hypothesis that enhanced excitability of SMA motor neurons contributes to DROSHA reduction via autophagy-lysosomal pathways.

### Reduced DROSHA enhances axonal growth in motor neurons

**Figure 7:**
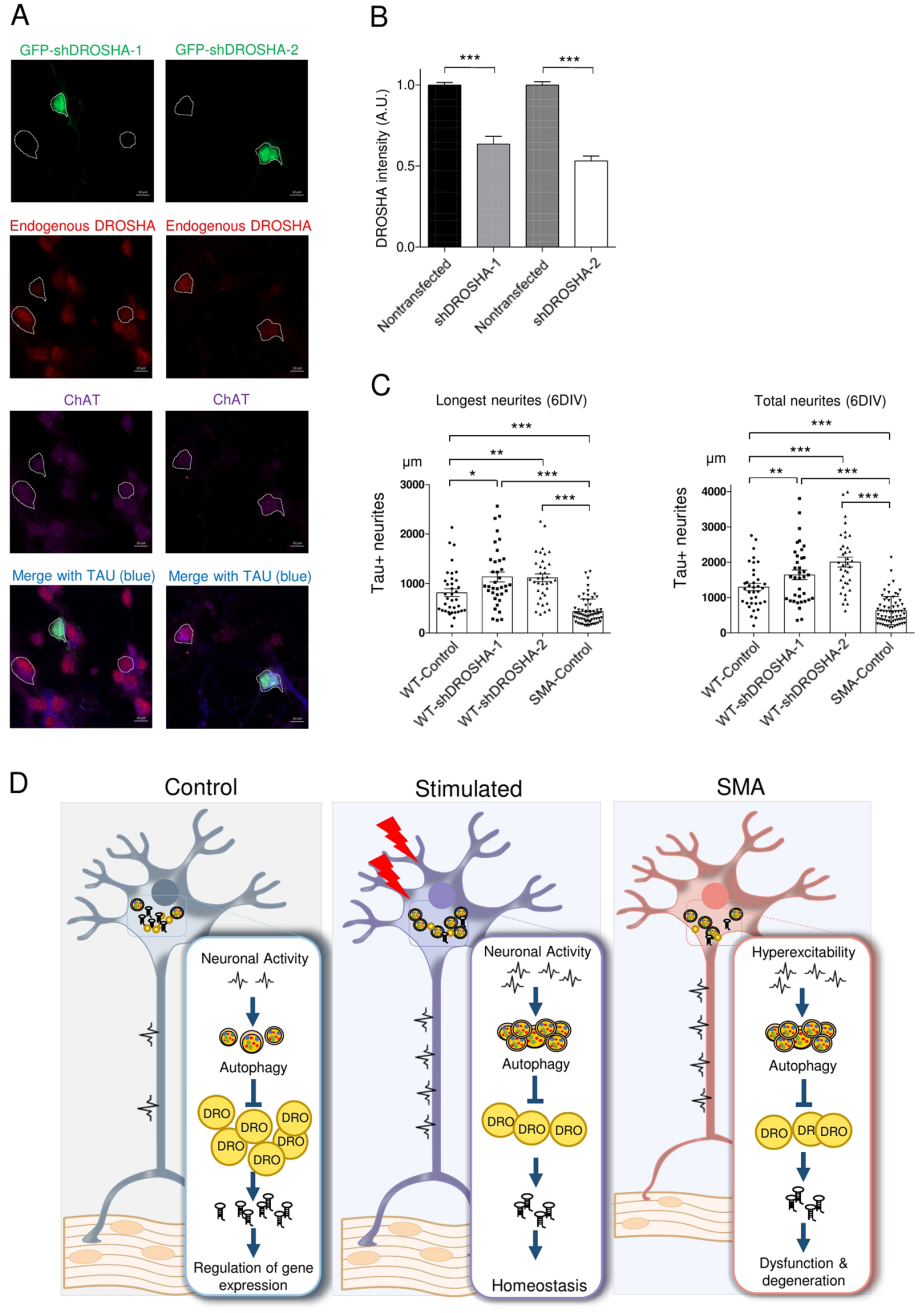
DROSHA reduction promotes neurites outgrowth. (**A**) Efficiency of two different shRNA sequences are tested. Plasmids were transfected in 1DIV and images were taken in 6DIV. DROSHA intensity from shRNA transfected neurons were compared to one from non-transfected neighboring neurons. GFP was used as a marker of transfection. (**B**) Bar graphs represent efficiency of shRNAs against *Drosha*. For shDROSHA-1, 18 transfected neurons are compared to 70 non-transfected neurons. For shDROSHA-2, 12 transfected neurons are compared to 42 non-transfected neurons. (**C**) Morphology of control and DROSHA knockdown motor neurons are visualized with GFP, and length of neurites was measured. Analyzed neurons were from 9 different transfections (from at least 3 independent biological samples). 36 neurons were analyzed per group. Analyzed neurons are all confirmed as ChAT positive (ChAT: Choline acetyltransferase, motor neuron marker). Data are represented as mean±SEM. Statistical significance is determined with t-test. *p < 0.05, **p < 0.01 and ***p < 0.001. (**D**) A schematic model for neuronal activity-autophagy-DROSHA-miRNA pathways in motor neurons.

We have found that DROSHA levels are reduced with neuronal activation in healthy WT motor neurons and in hyperexcitable SMA motor neurons. We then asked how reductions in DROSHA impacted motor neuron morphology. To answer this question, we knocked down *Drosha* with known shRNA in WT motor neurons^58^. To visualize the morphology of these neurons, we produced shRNA containing lentiviral vectors expressing GFP (named shDROSHA-1 and −2). To test shRNA efficiency, we transfected these plasmids to 1DIV motor neurons and measured the expression of DROSHA 5 days later. We confirmed that these shRNAs reduce DROSHA levels to ~50% of non-transfected neighbor neurons in the same culture (Fig. 7A,B). Next, we checked the morphology of *Drosha* KD motor neurons (ChAT positive). As our vectors express GFP, we used GFP signal together with TAU (a marker for axon) and ChAT (a marker for motor neuron) to visualize the morphology of transfected neurons. At 6DIV, we observed one dominant axon with a few small branches, while dendritic development was still at a minimum in control neurons (Supplementary Fig. 12). Interestingly, we found that reduction of DROSHA to 50% enhanced axonal growth (Fig. 7C, and Supplementary Fig. 13). However, consistent with previous reports^59^, inhibition of miR-218 impaired growth of motor neurons (Supplementary Fig. 14). Notably, further reduction of DROSHA (for example, 8DIV or more) seems to induce neuronal death (data not shown). Taken together, our data suggest that transient DROSHA reduction by autophagy promotes growth of neurons as a homeostatic response in healthy neurons, however, constant DROSHA reduction by hyperactivity/autophagy causes dysfunction and degeneration of SMA motor neurons (Fig. 7D).

## Discussion

Our findings indicate that neuronal activity enhances autophagy to decrease DROSHA/microRNA expression to promote neuronal growth in motor neurons and this pathway is pathologically altered in SMA neurons (Fig. 7D). As DROSHA is the core enzyme for miRNA biogenesis and miRNA dysregulation has been implicated in various neurological diseases including Alzheimer’s disease (AD) and ALS^8,60,61^, the mechanisms regulating *Drosha* expression including splicing, post-translational modification, and degradation have been intensively studied^19–23,44^. DROSHA levels are regulated by the mTOR-MDM2-proteasome pathway and the protease calpain in non-neuronal cells^21,22^. Surprisingly, while neurons express MDM2 and calpain, and both proteins play very important roles, DROSHA levels are instead regulated by autophagy in motor neurons. This finding suggests that molecular mechanisms regulating DROSHA expression are highly cell-type specific. Since DROSHA regulates the biogenesis of the majority of miRNAs, this implies that miRNA expression is regulated in a cell-type specific manner. Therefore, further studies need to examine whether DROSHA down-stream regulation including miRNAs and their target mRNAs are different among various types of cells.

DROSHA regulates the expression of motor neuron-specific miRNA, miR-218^59^. Loss of miR-218 causes neuromuscular failure, which is a hallmark of SMA. Here, we have demonstrated that miR-218 is reduced in SMA and *Drosha* KD motor neurons. Moreover, inhibition of miR-218 impaired growth of motor neurons. These findings raise the possibility that reductions of DROSHA and miR-218 contribute to neuromuscular phenotypes in SMA. In our previous work and current data, miR-183 levels are elevated in various *Smn* deficient cells. However, we observed instead a reduction in miR-183 expression in SMA motor neurons. The difference potentially can be explained by neuronal activity, as our previous data were obtained from either young neurons (6DIV) with severe SMN reduction or non-neuronal cells including fibroblasts and muscle cells. Moreover, transient *Smn* knockdown in 3DIV motor neurons did not change DROSHA levels. From these data, we can deduce that only mature SMA motor neurons with synaptic activity exhibit reductions of DROSHA as well as miR-183. This also supports the conclusion that DROSHA and miRNA-mediated gene expression are regulated cell type and context dependently.

Interestingly and surprisingly, our data show that the majority of miRNAs in motor neuron cultures are miR-10a and miR-10b. While it has been reported that miR-10 expression is caudal in human, mouse and zebrafish^45–47^, the role of miR-10 in post-mitotic motor neurons are not yet known. Furthermore, the biogenesis of both miRNAs are regulated by DROSHA and their amounts are reduced in SMA motor neurons, it will be important to further investigate the role of miR-10 families in healthy and degenerative motor neurons.

Although *Drosha* deletion can cause cellular dysfunction or even death (The Jackson Laboratory and^15,62^), certain levels of DROSHA reduction occur in non-pathological conditions such as depolarization or NMDA-mediated activation of neurons. We found that lowering DROSHA levels accelerates growth of neurons consistent with the fact that reduced DROSHA will lead to reduced functional miRNAs with subsequent disinhibition of mRNA translation. Our data suggest that neurons regulate *Drosha* expression to control cell growth; thus, DROSHA levels need to be controlled strictly by multiple layered mechanisms. In addition, we find that neuronal activity/autophagy reduces DROSHA level post translationally. This reduction might compensate for the phenotype of SMA motor neurons, in other words, this can be the homeostatic response of neurons against the disease pathology. However, further consistent reduction of DROSHA will cause dysregulation of many other miRNAs including miR-218, which may ultimately have detrimental effects on motor neurons. Together, it is noteworthy that certain levels of DROSHA reduction seem compensatory and beneficial, while too little DROSHA will cause cellular dysfunction and degeneration of neurons.

While DROSHA forms a complex with DGCR8, DGCR8 can bind to many other proteins including nucleolin and FUS RNA binding protein^63^. Like Drosha, DGCR8 can process mRNAs important for neurodevelopment^64^. As genetic deletion of DGCR8 in cortical progenitors impairs corticogenesis in a miRNA independent manner^64^ and dysregulated DGCR8/miRNA expression (increased or decreased) has been correlated with neurological disorders^65,66^, it will be interesting to test the role of elevated DGCR8 in motor neuron diseases.

Disturbed autophagy is described in the pathophysiology of numerous neurodegenerative disorders^67,68^. In neurodegenerative disorders that manifest with “protein aggregates” as cellular patho-phenotypes such as ALS and AD, inducing autophagy has been considered a promising therapy. However, the physiological role of autophagy as well as cell-type specific autophagy function needs to be carefully considered. The role of autophagy in motor neurons and in motor neuron disease is still not well understood. While mutations in genes important for autophagy function are associated with ALS in humans^69,70^, genetically modified mice with motor neuron specific inhibition of autophagy failed to recapitulate ALS^71^. These findings imply that regulation of autophagy is not simple and many pathways may compensate for each other to keep protein homeostasis in cells. In SMA, autophagy seems hyperactive and pharmacological inhibition of autophagosome formation moderates patho-phenotypes of an SMA mouse model^42,43^. Our data support these findings and revealed the additional molecular mechanism that hyperactive autophagy reduced the level of DROSHA complex causing dysregulation of RNA mediated gene expression in motor neurons. Taken together, understanding cell type specific gene expression is crucial to develop therapeutic tools for cell type specific disorders such as SMA.

## Materials and Methods

### Animal model

Primary cultured neurons were isolated from a Taiwanese SMA mouse model (FVB background)^72^. These mice were bred to produce in each litter 50% of offspring as SMA mice (*Smn*^KO/KO^;*SMN2*^tg/0^) and 50% phenotypically normal heterozygotes (*Smn*^KO/WT^;*SMN2*^tg/0^)^73^. Wild-type mice were used as controls (Jackson). Animal care and all surgical procedures were performed according to the institutional animal care committee guidelines and the German animal welfare laws, and approved under the reference numbers 84-02.05.20.13.042 and UniKoeln_Anziege§4.16.020 of the LANUV (Landesamt für Natur, Umwelt und Verbraucherschutz NRW) state agency of North-Rhine-Westphalia.

### Motor neuron culture

Primary motor neurons were isolated from E13.5 embryos. Spinal cords were dissociated in 1% Trypsin (Worthington) with DNase I (Applichem) via pipetting. Cells were seeded on Poly-D-Lysine (PDL, Sigma) coated plates/coverslips with neuronal plating media (Dulbecco’s modified Eagle’s medium supplemented with 5% fetal calf serum (FCS, Biochrom), 0.6% glucose, penicillin/streptomycin (Life Technologies) and amphotericin B (Promocell)). For imaging analysis, 75 K cells were plated per 12 well, and for protein analysis, 500 K cells were plated on 6-well plates. On the following day, neuronal plating media was replaced with motor neuron maintenance medium (Neurobasal medium (Life Technologies) supplemented with B27 supplement (Life Technologies), 2 mM L-glutamine, penicillin/streptomycin and amphotericin B with additional growth factors; ciliary neurotrophic factor (CNTF, 50 ng/ul, Peprotech), brain derived neurotrophic factor (BDNF, 50 ng/ul, Peprotech) and glia cell line derived neurotrophic factor (GDNF, 50 ng/ul, Peprotech)). Motor neurons were maintained at 37 °C in a humidified incubator with 5% CO_2_. One-half of media was changed each 3 days, and cytosine arabinoside (AraC) was added to a final concentration of 1 μM.

### Cortical neuron culture

Cortical neurons were isolated from E18 embryos. Briefly, cortices were dissected out and dissociated in neuronal plating media by pipetting. 400 K cells were seeded on PDL-coated 6-well plates with neuronal plating medium. On the following day, the neuronal plating media was replaced with Neurobasal medium supplemented with B27, 2 mM L-glutamine, penicillin/streptomycin and amphotericin B. Neurons were maintained at 37 °C in a humidified incubator with 5% CO_2_. One-half of media was changed each 3 days, and AraC was added to a final concentration of 1 μM.

### Protein isolation and Western blot analysis

Proteins were extracted with RIPA buffer (Sigma) supplemented with protease inhibitor cocktail (Roche) and protein concentration was determined by BCA assay (Thermo Scientific). Equal protein amounts were analysed with SDS–PAGE and immunoblotting according to a standard protocol. The information about antibodies can be found in Supplementary Table 1. Signals were detected with ChemiDoc XRS + System (BioRad), and quantification of signal was performed using Fiji software.

### Subcellular fractionation

Nucleus and cytoplasm were separated with Syn-PER Synaptic Protein Extraction Reagent (Thermo Scientific). Briefly, cells were lysed in 200 uL of Syn-PER Synaptic Protein Extraction Reagent, scraped off, collected, and centrifuged at 1,200 × g for 10 min at 4 °C. After the centrifugation, the supernatant containing the cytoplasm was separated from the pellet. The pellet was then lysed in 200 uL of RIPA buffer, and incubated on ice for 10 min. After this, the samples were centrifuged at 12,000 × g for 10 min at 4 °C. The supernatant was transferred to a new tube (nuclear fraction). Protease inhibitor cocktail (Roche) and phosphatase inhibitor cocktail (Thermo Scientific) were added to Syn-PER reagent and RIPA buffer before use.

### shRNA containing plasmids construction

We constructed lentiviral vectors containing shRNAs against mouse *Drosha*. We used two published shRNA sequences (shDROSHA-1 and shDROSHA-2)^58^. These vectors also express GFP to visualize morphology of neurons.

### Immunostaining of motor neurons

Motor neurons were grown on PDL coated cover-slips for 10 days. Neurons were fixed with 4% paraformaldehyde (PFA) supplemented with 4% sucrose, at room temperature (RT) for 20 min. Neurons were permeabilized with 0.2% PBS-T at RT for 15 min. Subsequently, neurons were incubated in 3% BSA (in 0.2% PBS-T) at RT for 1 hour, and incubated with primary antibodies at 4 °C overnight. Next day, neurons were washed, and incubated with secondary antibodies at RT for 3 hours, washed again, and the nucleus was stained with DAPI (Thermo Scientific). Finally, coverslips were mounted on glass slides with Mowiol (Sigma). The information about antibodies can be found in Supplementary Table 1.

### Measuring axon length (*Drosha* KD neurons)

100,000 motor neurons were seeded on PDL coated cover-slips in 12 well plates in plating media. A day after seeding shRNA containing plasmids were transfected, and culture media was changed to maintenance media. After 5 days, neurons were fixed and stained with TAU and ChAT as described above. Pictures of GFP, TAU and ChAT positive neurons were taken with the fluorescence microscope (Axio imager M2, Zeiss) and length of neurites was measured by Fiji (Example pictures in Supplementary Fig. 13).

### Image analysis

All images were acquired with fluorescence Zeiss microscope (Axio Imager.M2) equipped with an AxioCam MR camera and an ApoTome.2 system (Institute of Human Genetics, University of Cologne). Images were analyzed with the ZEN (Zeiss) or Fiji.

### RNA isolation, cDNA synthesis and real time PCR

Total RNA was extracted from neurons using the *mirVana™* miRNA Isolation Kit (Thermo Scientific). We followed the manufacturer’s instructions. RNA concentration was determined using the NanoDrop ND-1000 spectrophotometer (Peqlab). cDNA was produced from total RNA using the High-Capacity cDNA Reverse Transcription Kit (Thermo Scientific) with random primers. mRNA expression was determined by real-time PCR with PowerSYBR® Green PCR Master Mix (Thermo Scientific) and 1 μM of gene specific primers. The amplification conditions for *Drosha* and *Actb* were: an initial incubation stage at 50 °C for 2 min, denaturation at 95 °C for 10 min and 40 cycles of amplification step (95 °C for 15 s, 60 °C for 30 s, and 72 °C for 40 s). For the amplification of *Dgcr8*, the same conditions were used except using an annealing temperature of 65 °C. An additional dissociation step was added to confirm the correct amplification and PCR product was confirmed with Sanger sequencing. Real time PCR was performed with 7500 Real-Time PCR System (Thermo Scientific). Sequences of gene specific primers are listed in Supplementary Table 2.

### miRNA sequencing

1 ug of total RNA per sample was used for library construction, and sequencing was performed in the Cologne Center for Genomics. Detailed methods were described previously^74^.

### Real time PCR for primary, precursor, and mature microRNA assays

In order to quantify primary and mature miRNA levels, TaqMan MicroRNA assay was used (Applied Biosystems). For precursor miRNA assay, we used miScript Precursor assay with miScript SYBR Green PCR kit (Qiagen). We used 50 ng of total RNA per reaction and followed the manufacturer’s instructions. The information about miRNA expression assays used for this study can be found in Supplementary Table 3.

### Drug treatment

Motor neurons were treated with various drugs. The drugs used in this study are TTX (T8024, Sigma), riluzole (R116, Sigma), 3-methyladenine (3-MA, M9281, Sigma), MG132 (M7449, Sigma), nutlin3 (N6287, Sigma), calpeptin (C8999, Sigma) and bafilomycin A (B1793, Sigma), N-methyl D-aspartate (NMDA, Cay-14581, Biomol), rapamycin (#9904, Cell Signaling), Torin-1 (#CALB475991, Calbiochem), 10-NCP (#2338, Biovision) and cycloheximide (#01810, Sigma). Concentrations of drugs used in this study can be found in the figure legends.

### siRNAs, miRNA inhibitors and shRNA transfection

The knockdown of *Drosha* was performed using siRNA technology (FlexiTube, Qiagen). Two different siRNAs for *Drosha* (Mn_Ethohi2_1 #SI00996499 and Mn_Ethohi2_3 #SI00996513) or negative control siRNA (#1027280) were transfected with Lipofectamin®2000 (Thermo Scientific). For *Atg5* and *Smn* knockdown, we transfected siRNAs (*Atg5* s62452; *Smn* s133926 or negative control 4390843, *Silencer® Select* Pre-designed siRNA, Thermo Scientific) with Lipofectamin®2000. To inhibit miR-218 activity, miR-218 inhibitor (AM10328, Thermo Scientific) and control (AM17010, Thermo Scientific) were transfected with Lipofectamine®3000 (Thermo Scientific). For transfection of shRNA containing plasmid, we used 1 ug of DNA with 2 ug of Lipofectamin®3000 per coverslip. We mostly followed the manufacturer’s instructions, however, we replaced the culture media 1 hour after transfection for WT motor neurons, while we changed the media 45 min after transfection for SMA motor neurons.

### PCR for *Drosha* splicing variants

To check Drosha splicing variants in exon 7 region in WT and SMA motor neurons, we performed PCR assay. We designed specific primers to amplify the region between exon 5 to exon 8 based on the sequence of *Mus musculus Drosha* transcripts (GenBank, NM_001130149.1). The PCR condition was 94 °C for 3 min followed by 39 cycles of amplification (94 °C for 45 sec, 64 °C for 30 sec and 72 °C for 1 min), and final extension at 72 °C for 5 min. PCR products were confirmed by Sanger sequencing. Primer sequences used for this experiments are listed in Supplementary Table 4.

### Data availability statement

miRNA sequencing data can be found in the supplementary dataset. The datasets generated and analyzed during the current study are available from the corresponding author on reasonable request.

## Electronic supplementary material

Supplementary information

Supplementary datasest
