# Author Correction: Neuronal activity regulates DROSHA via autophagy in spinal muscular atrophy

**DOI:** 10.1038/s41598-018-28202-6

**Published:** 2018-07-03

**Authors:** Inês do Carmo G. Gonçalves, Johanna Brecht, Maximilian P. Thelen, Wiebke A. Rehorst, Miriam Peters, Hyun Ju Lee, Susanne Motameny, Laura Torres-Benito, Darius Ebrahimi-Fakhari, Natalia L. Kononenko, Janine Altmüller, David Vilchez, Mustafa Sahin, Brunhilde Wirth, Min Jeong Kye

**Affiliations:** 1 Institute of Human Genetics, University of Cologne, Cologne, 50931 Germany; 2 Center for Molecular Medicine Cologne, University of Cologne, Cologne, 50931 Germany; 3 Institute for Genetics, University of Cologne, Cologne, 50931 Germany; 4 Cologne Excellence Cluster for Cellular Stress Responses in Aging-Associated Diseases (CECAD), University of Cologne, 50931 Cologne, Germany; 5 Cologne Center for Genomics (CCG), University of Cologne, 50931 Cologne, Germany; 6 Department of Neurology, The F.M. Kirby Center for Neurobiology, Boston Children’s Hospital, Harvard Medical School, Boston, MA 02115 USA; 7 Center for Rare Diseases, University Hospital Cologne, Cologne, Germany

Correction to: *Scientific Reports* 10.1038/s41598-018-26347-y, published online 21 May 2018

The Acknowledgements section in this Article is incomplete.

“We thank to Dr. Leo Kurian and Dr. Peter Tsai for critical reading of the manuscript. We furthermore thank the Regional Computing Center of the University of Cologne (RRZK) for providing computing time on the DFG-funded HPC cluster CHEOPS as well as support. This work is supported by German Research Foundation (KY96/1–1 to MK, KO5091/2-1 to NLK, Wi-945/14-1 and Wi 945/17-1 to BW), Cologne Fortune (MK), CureSMA (MK and MS), SMA Europe (MK), CECAD Exc-229 (NLK), AFM-Telethon (LTB), CMMC (BW) and EU FP7 NEUROMICS (BW).”

should read:

“We thank to Dr. Leo Kurian and Dr. Peter Tsai for critical reading of the manuscript. We furthermore thank the Regional Computing Center of the University of Cologne (RRZK) for providing computing time on the DFG-funded HPC cluster CHEOPS as well as support. This work is supported by German Research Foundation (KY96/1-1 to MK, KO5091/2-1 to NLK, Wi-945/14-1 and Wi 945/17-1 to BW), Cologne Fortune (MK), CureSMA (MK and MS), SMA Europe (MK), Slaney Family Fund (MS), CECAD Exc-229 (NLK), AFM-Telethon (LTB), CMMC (BW) and EU FP7 NEUROMICS (BW).”

