# Author Correction: Neuronal activity regulates DROSHA via autophagy in spinal muscular atrophy

**DOI:** 10.1038/s41598-020-64694-x

**Published:** 2020-05-13

**Authors:** Inês do Carmo G. Gonçalves, Johanna Brecht, Maximilian P. Thelen, Wiebke A. Rehorst, Miriam Peters, Hyun Ju Lee, Susanne Motameny, Laura Torres-Benito, Darius Ebrahimi-Fakhari, Natalia L. Kononenko, Janine Altmüller, David Vilchez, Mustafa Sahin, Brunhilde Wirth, Min Jeong Kye

**Affiliations:** 1 Institute of Human Genetics, University of Cologne, Cologne, 50931 Germany; 2 Center for Molecular Medicine Cologne, University of Cologne, Cologne, 50931 Germany; 3 Institute for Genetics, University of Cologne, Cologne, 50931 Germany; 4 Cologne Excellence Cluster for Cellular Stress Responses in Aging-Associated Diseases (CECAD), University of Cologne, 50931 Cologne, Germany; 5 Cologne Center for Genomics (CCG), University of Cologne, 50931 Cologne, Germany; 6 Department of Neurology, The F.M. Kirby Center for Neurobiology, Boston Children’s Hospital, Harvard Medical School, Boston, MA 02115 USA; 7 Center for Rare Diseases, University Hospital Cologne, Cologne, Germany

Correction to: *Scientific Reports* 10.1038/s41598-018-26347-y, published online 21 May 2018

This Article contains an error. In Figure 3C, the panels for tau treated wild-type and spinal muscular atrophy motor neurons are reversed. The correct Figure 3C appears below as Figure 1.

**Figure 1:**
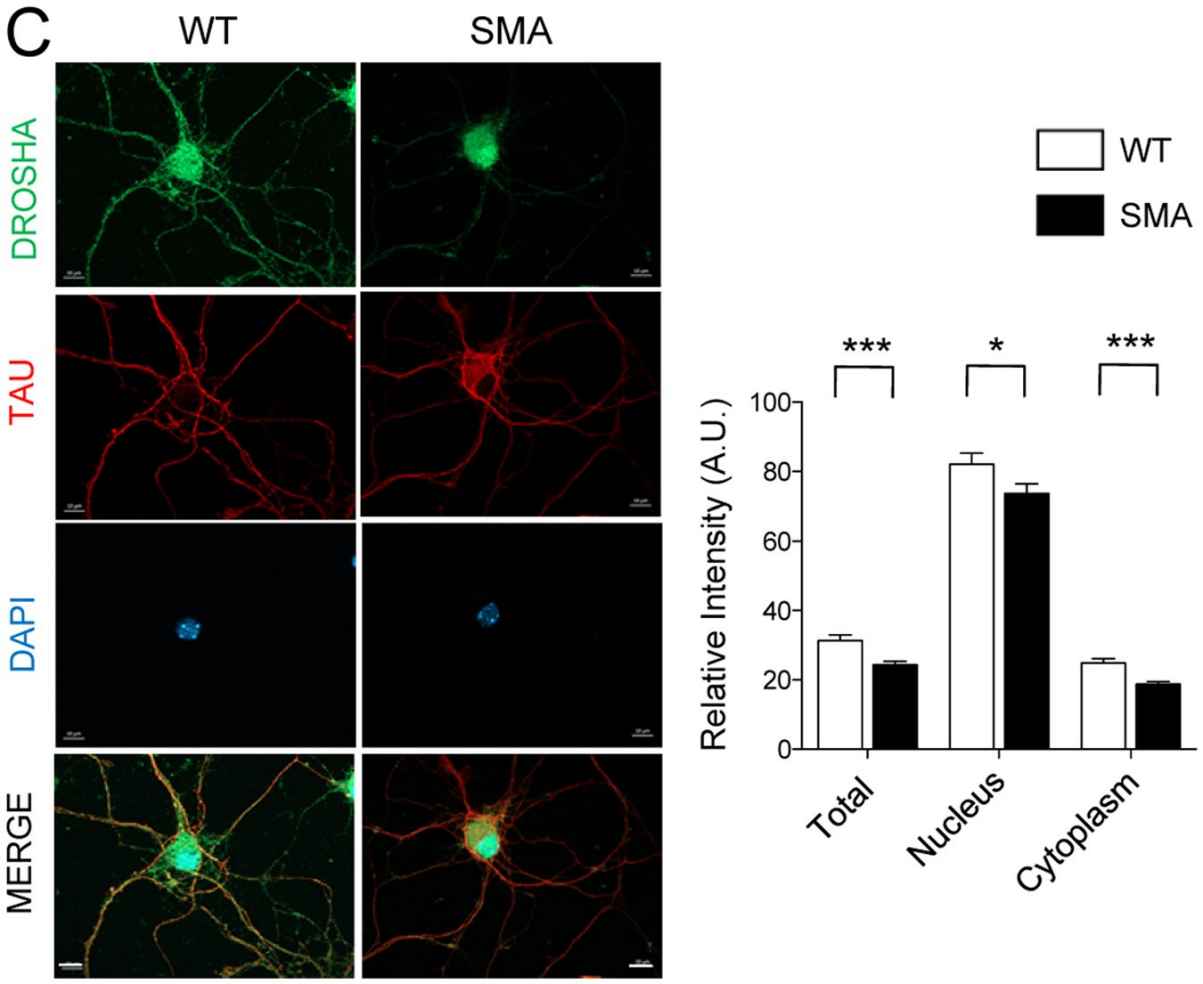
.

