# Supplementary material for: Neuronal activity regulates DROSHA via autophagy in spinal muscular atrophy
Source: Sci Rep. 2018 May 21;8:7907. doi: 10.1038/s41598-018-26347-y (PMC5962575; doi:10.1038/s41598-018-26347-y)

Supplementary Figures and Tables

Neuronal activity regulates DROSHA via autophagy in spinal muscular atrophy

Inês do Carmo G. Gonçalves[1], Johanna Brecht[1], Maximilian P. Thelen[1], Wiebke A. Rehorst[1], Miriam Peters[1,2], Hyun Ju Lee[3,4], Susanne Motameny[5], Laura Torres-Benito[1,2], Darius Ebrahimi-Fakhari[6], Natalia L. Kononenko[4], Janine Altmüller[1,2,5], David Vilchez[3,4], Mustafa Sahin[6], Brunhilde Wirth[1,2,3,7] and Min Jeong Kye[1*]

[1]Institute of Human Genetics, University of Cologne, Cologne, 50931, Germany; [2]Center for Molecular Medicine Cologne, University of Cologne, Cologne, 50931, Germany; [3]Institute for Genetics, University of Cologne, 50931, Germany; [4]Cologne Excellence Cluster for Cellular Stress Responses in Aging-Associated Diseases (CECAD), University of Cologne, 50931 Cologne, Germany. [5]Cologne Center for Genomics (CCG), University of Cologne, 50931, Cologne, Germany; [6]Department of Neurology, The F.M. Kirby Center for Neurobiology, Boston Children's Hospital, Harvard Medical School, Boston, MA, 02115, USA and [7]Center for Rare Diseases, University Hospital Cologne, Cologne, Germany

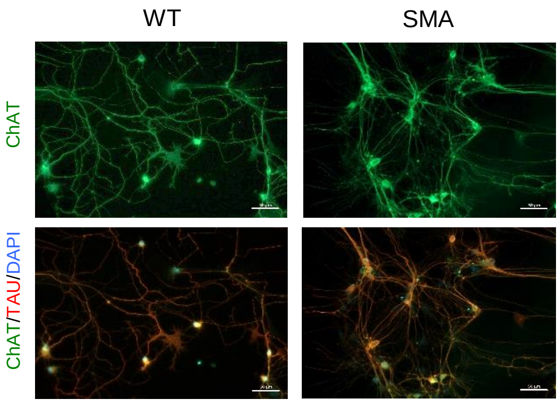

Supplementary Fig. 1. Images display expression of the motor neuron marker, choline acetyltransferase (ChAT, green) and neuronal marker TAU (red) in 10 days *in vitro* (10DIV) primary motor neurons. DAPI (nucleus, blue), Scale bar: 50µm

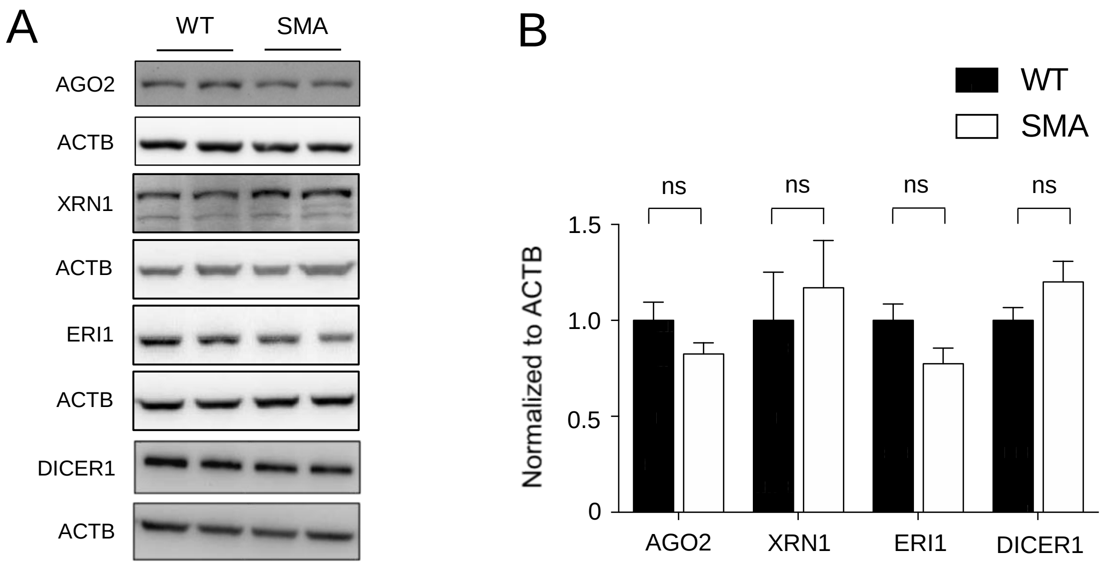

Supplementary Fig. 2. Representative Western blots (A) and quantification (B) of AGO2, XRN1, ERI1 and ACTB in 10DIV motor neurons from WT and SMA mice. Each sample represents an individual embryo. At least 4 embryos per genotype were tested (for AGO2 and XRN1: n=4 per group, for ERI1: n=12 per group, and for DICER1: n=9 per group). Data are represented as mean ± SEM. Statistical difference was tested with t-test, and there was no significant difference between WT and SMA. ns=not significant

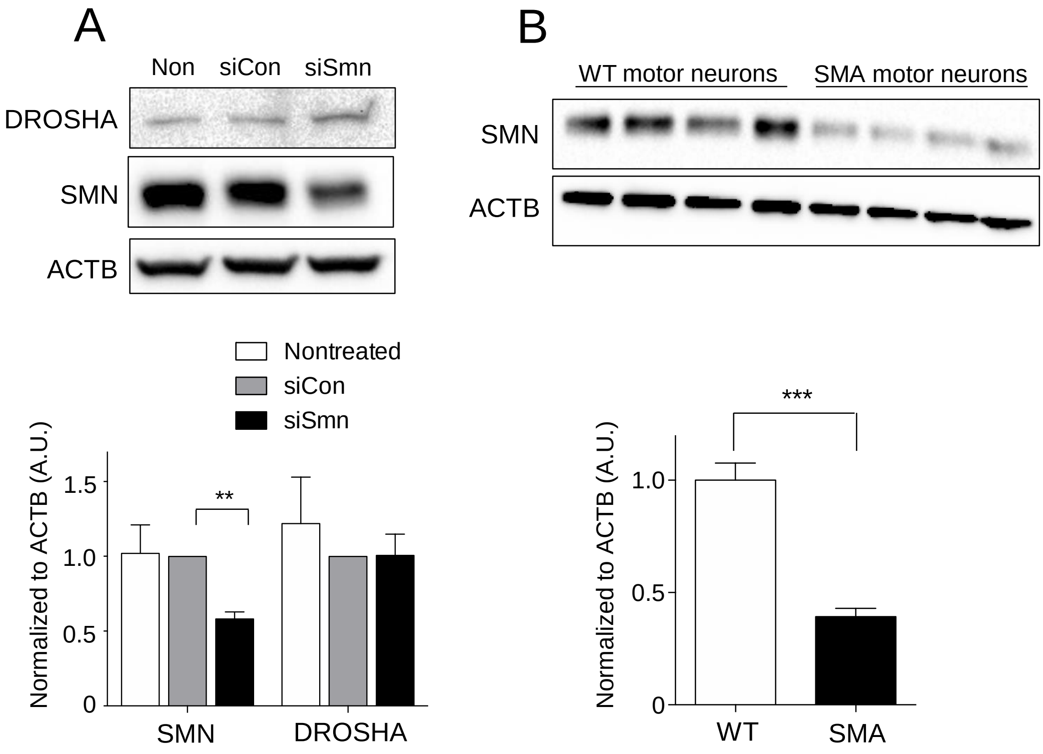

Supplementary Fig. 3. Transient *Smn* knockdown did not change DROSHA expression in WT motor neurons. (A) Control or siRNA against *Smn* were transfected to 3DIV WT motor neurons (50pmol). Knockdown (KD) efficiency was confirmed by Western blot after 72h of transfection. Bar graph summarizes results from 4 independent experiments. Protein levels were normalized to ACTB, and represented as relative levels compared to control siRNA (siCon) transfected samples. (B) SMN levels were measured in 10DIV WT and SMA motor neurons. N=4 per group. SMN levels were normalized to ACTB, and represented as relative levels compared to WT ones. Data are represented as mean ± SEM. Statistical significance was determined with t-test. **p<0.01, ***p<0.001.

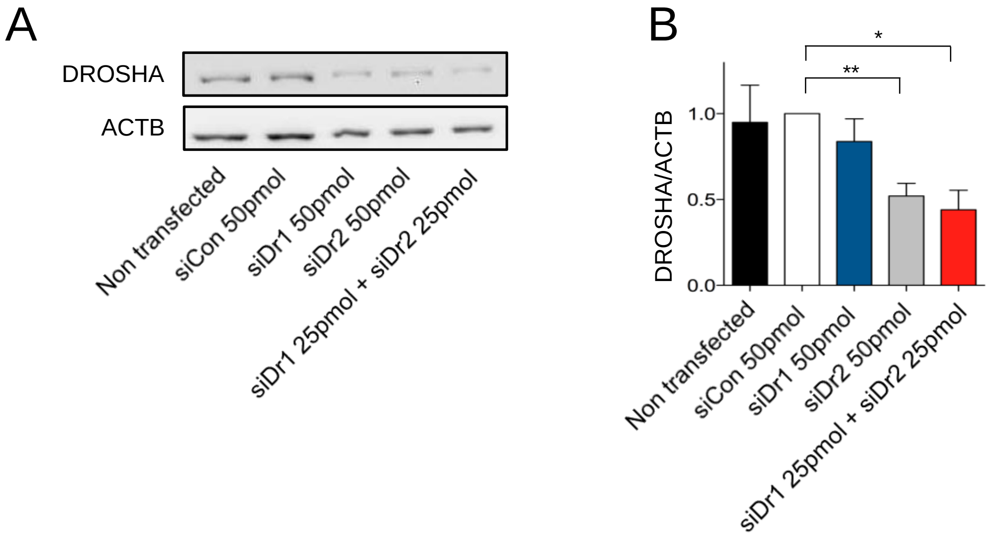

Supplementary Fig. 4. (A) Optimization of *Drosha* knockdown (KD) in WT cortical neurons. Control (siCon) or two different siRNAs against *Drosha* (siDr1 and siDr2) were transfected to 3DIV cortical neurons individually (50pmol) or combined (25pmol each). KD of *Drosha* was confirmed by Western blot after 72h of transfection. (B) Bar graph summarizes results from 4 independent experiments. DROSHA levels were normalized to ACTB, and represented as relative values compared to control siRNA (siCon) transfected samples. Data are represented as mean ± SEM. Statistical significance was determined with t-test. *p<0.05 and **p<0.01.

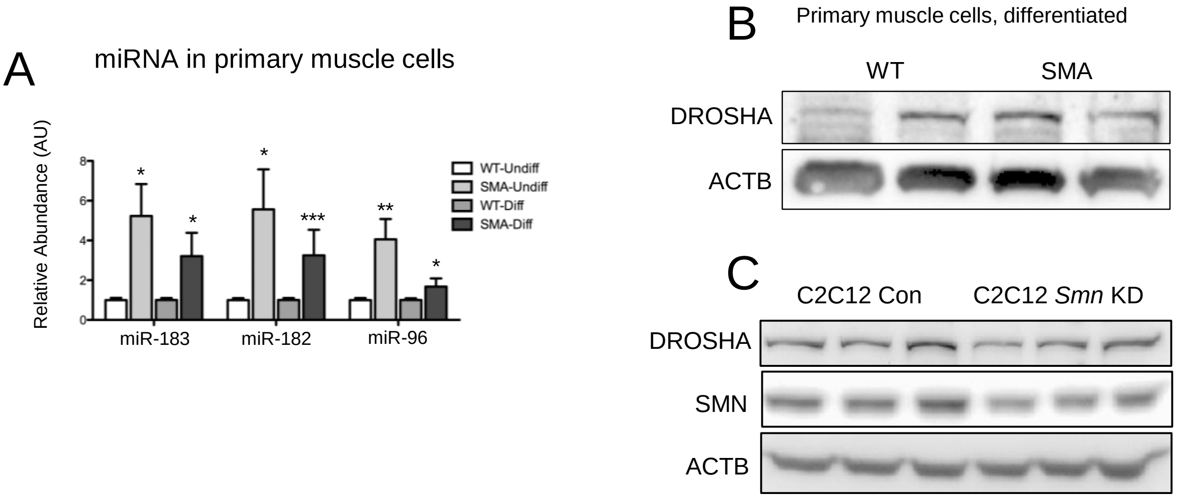

Supplementary Fig. 5. (A) Expression of individual miRNAs in miR-183 cluster was measured with quantitative RT-PCR in muscle cells isolated from WT and SMA mice. We measured miRNAs in undifferentiated (myoblasts and mainly myocytes) and differentiated (mainly myotube) culture. n=24 for WT- and SMA-undifferentiated, n=34 for WT and SMA differentiated muscle cells. (B) Western blot showing DROSHA levels in differentiated muscle cells isolated from WT and SMA mice. (C) Western blot showing DROSHA levels in differentiated control and *Smn* knockdown C2C12 cells. *Smn* was knocked down with siRNAs. Reduced SMN level was confirmed with Western blot. Data are represented as mean ± SEM. Statistical significance was determined with t-test. *p<0.05, **p<0.01 and ***p<0.001.

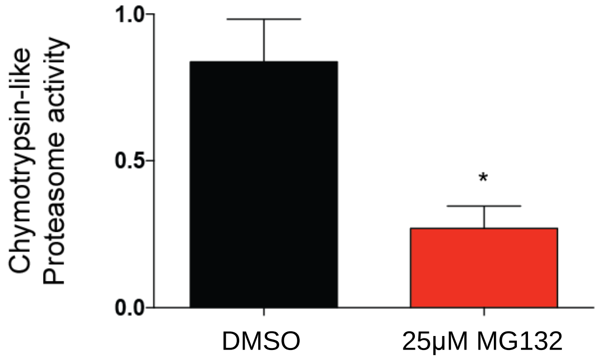

Supplementary Fig. 6. Proteasome assay confirms that 25μM MG132 treatment successfully inhibits proteasome activity in motor neurons. 25μM MG132 was treated to 9DIV WT motor neurons for 24 hours. n=3 from three independent samples. Data are represented as mean ± SEM. Statistical significance was determined with t-test. *p<0.05.

Methods

26S proteasome fluorogenic peptidase assay

In vitro assay of 26S proteasome activity was performed as previously described in(**REF**). Neurons were collected in proteasome activity assay buffer (50mM Tris-HCl, pH7.5, 250mM sucrose, 5 mM MgCl2, 0.5 mM EDTA, 2 mM ATP and 1 mM dithiothreitol) and lysed by passing 10 times through a 27G needle attached to a 1 ml syringe needle. Then, neuronal lysates were centrifuged at 10,000g for 10 min at 4°C and supernatants were collected. 25 μg of protein were transferred to a 96-well microtiter plate (BD Falcon) and incubated with the fluorogenic proteasome substrate Z-Gly-Gly-Leu-AMC (Enzo). Fluorescence (380 nm excitation, 460 nm emission) was monitored on a microplate fluorometer (EnSpire, Perkin Elmer) every 5 min for 1 h at 37°C.

Reference: Kisselev, A. F. & Goldberg, A. L. Monitoring activity and inhibition of 26S proteasomes with fluorogenic peptide substrates. *Methods Enzymol* **398**, 364-378, (2005).

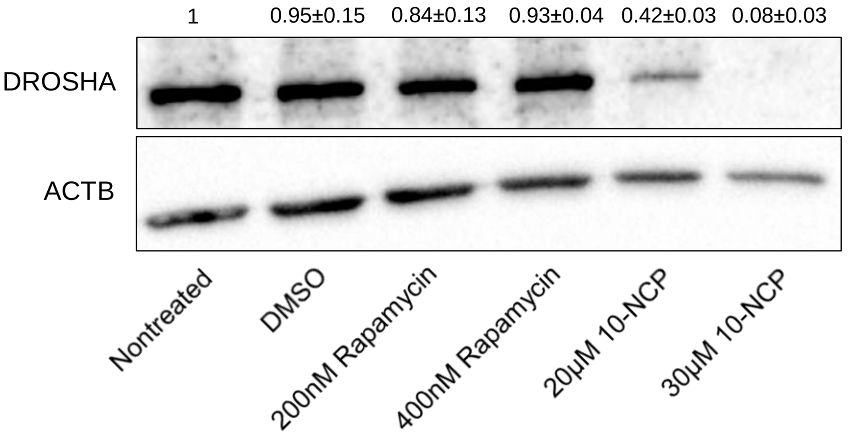

Supplementary Fig. 7. Higher concentrations of autophagy inducer, 10-NCP reduced DROSHA levels. 10DIV WT motor neurons were treated with various concentrations of autophagy inducers, rapamycin and 10-NCP for 24 hours. 10-NCP reduced DROSHA levels dose-dependently. Notably, 200nM or 400nM rapamycin did not reduce DROSHA levels in WT motor neurons. Data are from two independent experiments. Mean ± SEM.

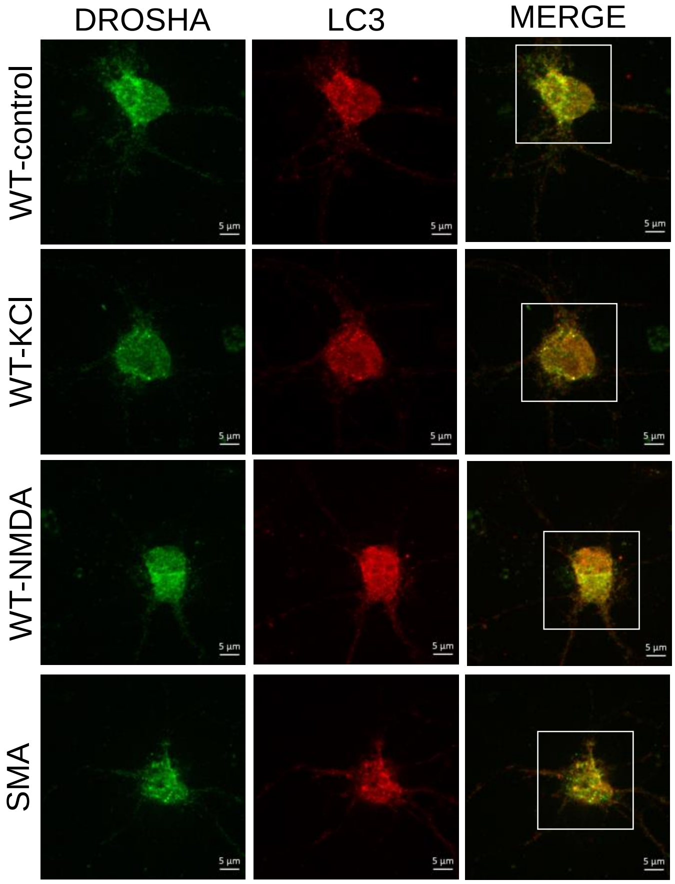

Supplementary Fig. 8. Single channel and merged pictures of DROSHA and autophagosome marker LC3 in WT and SMA motor neurons. 10DIV WT motor neurons were stimulated with 55mM KCl or 100μM NMDA for 1 hour. SMA motor neurons were not stimulated. Green: DROSHA, Red: LC3. Squares represent the area in Fig. 6A. Scale bar=5 μm.

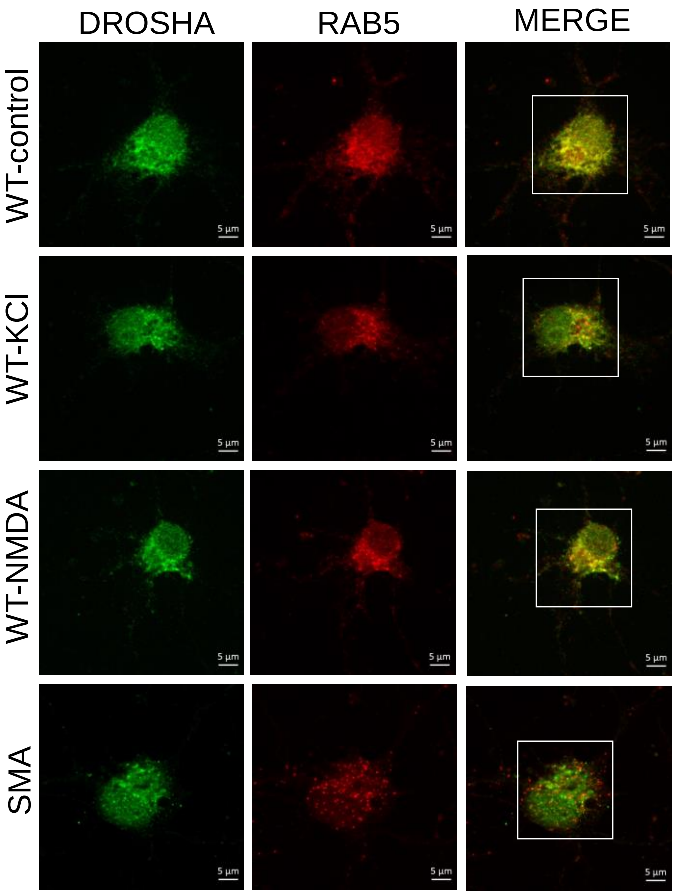

Supplementary Fig. 9. Single channel and merged pictures of DROSHA and RAB5 in WT and SMA motor neurons. 10DIV WT motor neurons were stimulated with 55mM KCl or 100μM NMDA for 1 hour. SMA motor neurons were not stimulated. Green: DROSHA, Red: RAB5. Squares represent the area in Fig. 6A. Scale bar=5 μm.

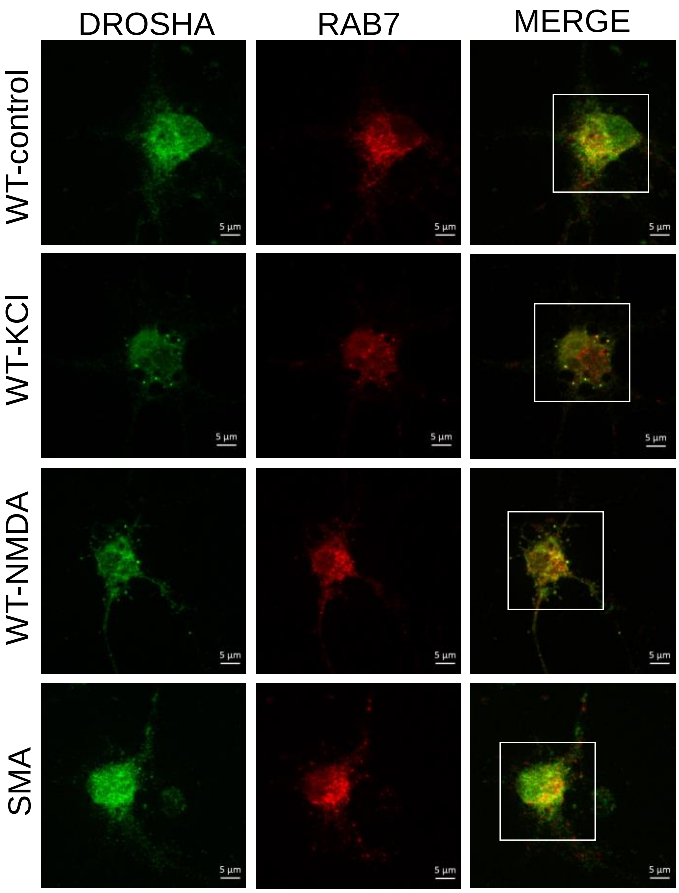

Supplementary Fig. 10. Single channel and merged pictures of DROSHA and RAB7 in WT and SMA motor neurons. 10DIV WT motor neurons were stimulated with 55mM KCl or 100μM NMDA for 1 hour. SMA motor neurons were not stimulated. Green: DROSHA, Red: RAB7. Squares represent the area showing in Fig. 6A. Scale bar=5 μm.

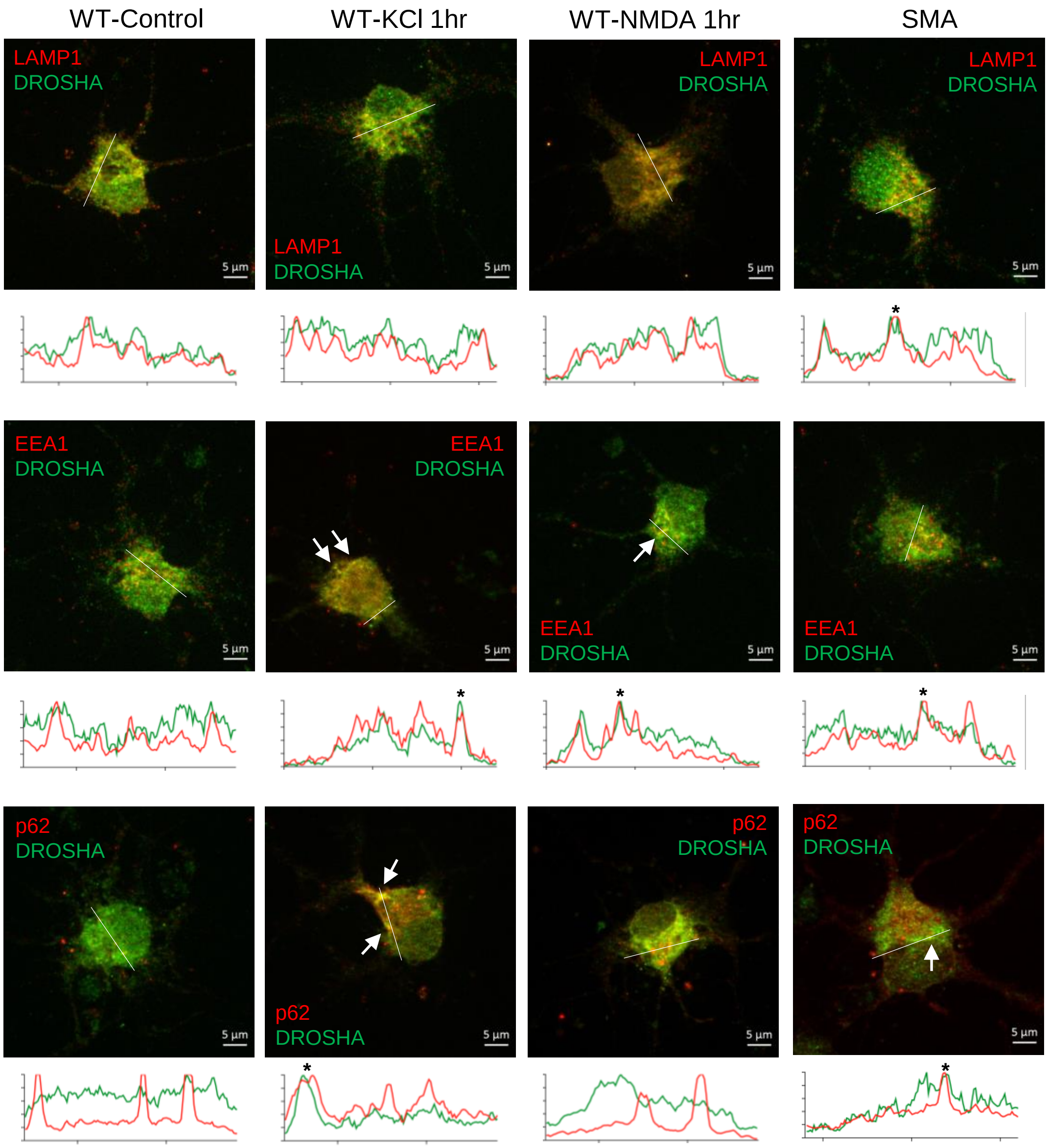

Supplementary Fig. 11. DROSHA localization with vesicular markers in WT and SMA motor neurons. DROSHA signals were not colocalized with LAMP1 signals, and partially localized with EEA1 and p62 signals in stimulated or SMA 10DIV motor neurons. Neurons were treated with 55mM KCl or 100μM NMDA for 1 hour. Green: DROSHA, red: vesicular markers, listed in figure.
*; overlapped signals. Scale bar=5 μm.

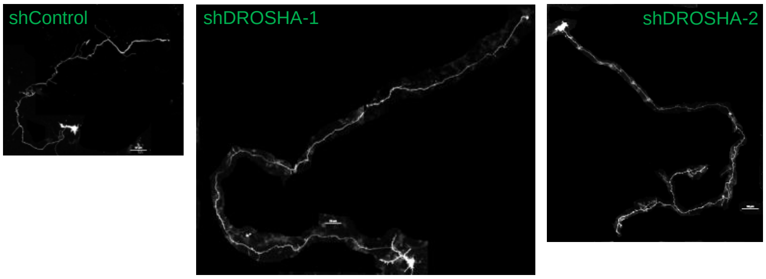

Supplementary Fig. 12. Morphology of Control and *Drosha* knockdown motor neurons. Plasmids containing shRNA against Drosha was transfected to 3DIV WT motor neurons, and morphology was checked in 72 hours later (in 6DIV). shRNA containing plasmids also express GFP, thus transfected neurons can be visualized. Images were taken in same magnification. Scale bar=50 μm.

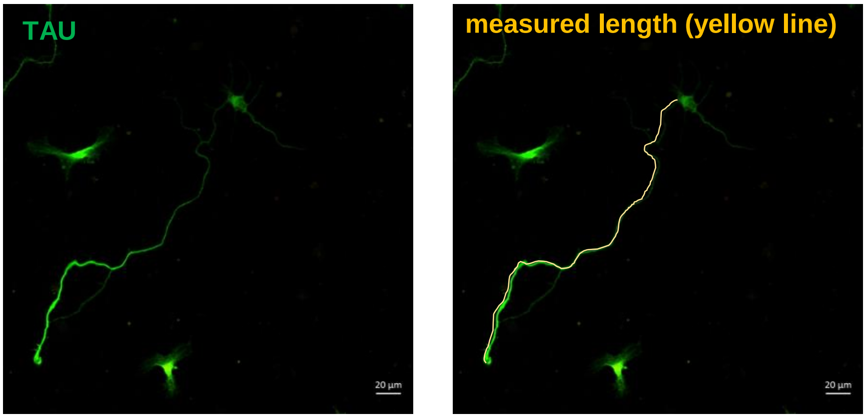

Supplementary Fig. 13. Representative images showing how we measure axon length. First, we visualize neuronal morphology with Tau or GFP. Then, Tau positive or GFP positive neurites (TAU for axons) were measured with Fiji software. We have measured the length from starting point of axons (end of somata) till end of axons (growth cones). Scale bar=20 μm.

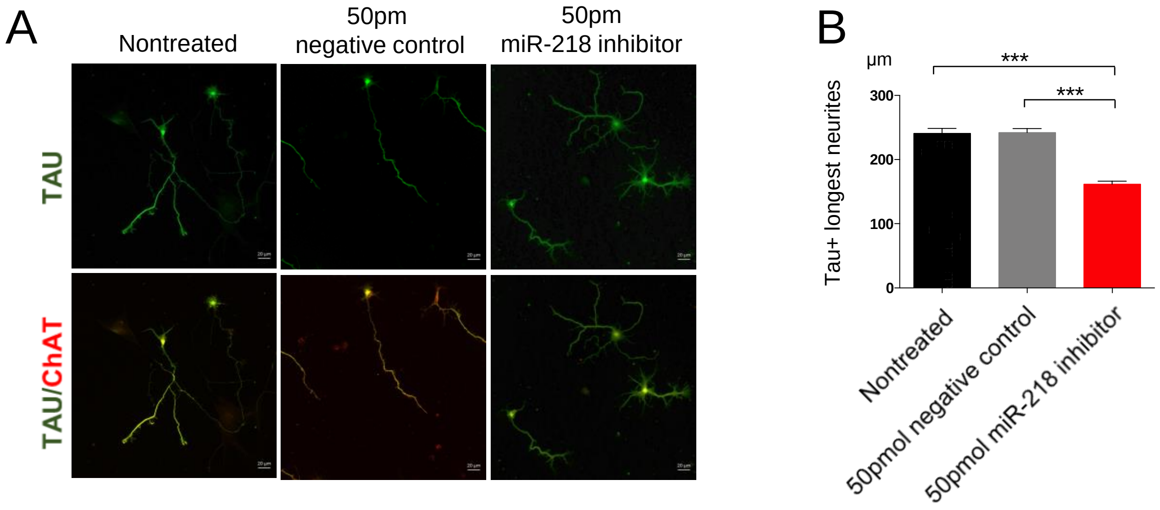

Supplementary Fig. 14. miR-218 inhibitor impairs neurites outgrowth in 3-5 DIV WT motor neurons. Low density of spinal motor neurons (75K for 12 wells) were transfected with negative control or miR-128 inhibitor (50pmol) in 3DIV, and length of TAU positive neurites from ChAT positive neurons were measured after 48 hours. (A) Representative images of motor neurons, non-treated, treated with 50pmol negative control or 50pmol miR-218 inhibitor. Green:TAU, Red:ChAT (B) The length of TAU positive longest neurites was reduced in miR-218 inhibitor treated motor neurons. N=189 (non-treated), n=222 (negative control), n=272 (miR-218 inhibitor) from each 7 coverslips. Neurons are from at least three independent experiments. Data are represented as mean ± SEM. Statistical significance was determined with Student's t-test, ***p<0.001, scale bar = 20μm

Supplementary Tables

Supplementary Table 1. Antibodies used in this study

Antibody	#Catalogue	Manufacturer	Dilution factors and applications
DROSHA (D28B1)	3364	Cell Signaling	WB 1:1000<br>IF 1:800
DGCR8	SAB4200089	Sigma	WB 1:500
DICER1	SAB4200087	Sigma	WB 1:500
XRN1 (K-20)	sc-50209	Santa Cruz	WB 1:350
TAU (A10)	sc-390476	Santa Cruz	IF 1:800
TAU	Ab75714	Abcam	IF 1:200
LC3B	L7543	Sigma	WB 1:1000<br>IF 1:500
p62/SQSTM1	P0067	Sigma	WB 1:1000
p62/SQSTM1	Ab56416	Abcam	IF 1:200
RAB7 (B-3)	sc-376362	Santa Cruz	IF 1:200
Lamp-1 (H4A3)	sc-20011	Santa Cruz	IF 1:200
RAB5 (D-11)	sc-46692	Santa Cruz	IF 1:200
Lamin B1 (A-11)	sc-377000	Santa Cruz	WB 1:800
EEA1	610456	BD Transduction	IF 1:50
ChAT	AB144P	Millipore	IF 1:250
ACTB-HRP (C4)	sc-47778	Santa Cruz	WB 1:1000
ATG5	12994	Cell Signaling	WB 1:1000
AGO2	sc-376696	Santa Cruz	WB 1:200

WB: Western blot IF: Immunofluorescence

Supplementary Table 2. Primer sequences used for real-time qPCR.

Primers	Sequence 5’-3’
ACTB-F-469	AGCCATGTACGTAGCCATCC
ACTB-R-696	CTCTCAGCTGTGGTGGTGAA
mDrosha-F	GGACCATCACGAAGGACACT
mDrosha-R	GATGTACAGCGCTGCGATAA
mDGCR8-F	GCTGCAGGAGTAAGGACAGG
mDGCR8-R	TCGAGCACTGCATACTCCAC

Supplementary Table 3. microRNA assays

miRNAs	#Catalogue
miR-218	TM000521 (Thermo Fisher Scientific)
miR-183	TM002269 (Thermo Fisher Scientific)
miR-128a	TM002216 (Thermo Fisher Scientific)
miR-26a	TM000405 (Thermo Fisher Scientific)
miR-25	TM000403 (Thermo Fisher Scientific)
miR-19a	TM000395 (Thermo Fisher Scientific)
miR-182	TM002334 (Thermo Fisher Scientific)
miR-96	TM000434 (Thermo Fisher Scientific)
miR-877*	TM002548 (Thermo Fisher Scientific)
miR-10a	TM000387 (Thermo Fisher Scientific)
miR-10b	TM002218 (Thermo Fisher Scientific)
Pri-miR-218-1	Mm03307136-pri (Thermo Fisher Scientific)
Pri-miR-218-2	Mm03307143-pri (Thermo Fisher Scientific)
Pri-miR-183	Mm03306888-pri (Thermo Fisher Scientific)
Pre-miR-218-1	MP00005159 (Qiagen)
Pre-miR-218-2	MP00005166 (Qiagen)
Pre-miR-183	MP00004438 (Qiagen)

Supplementary Table 4. Primer sequences for *Drosha* splicing variants

Primers	Target region	Sequences 5’-3’
Drosha E5-F16	exon 5- exon 8	CTCAAGCACTACGACGACCA
Drosha-E8-R1	exon 5- exon 8	CTCCTCCTCCTCCGTCTTCT

Original Western blot images for Fig. 1

DROSHA (Fig 1A)

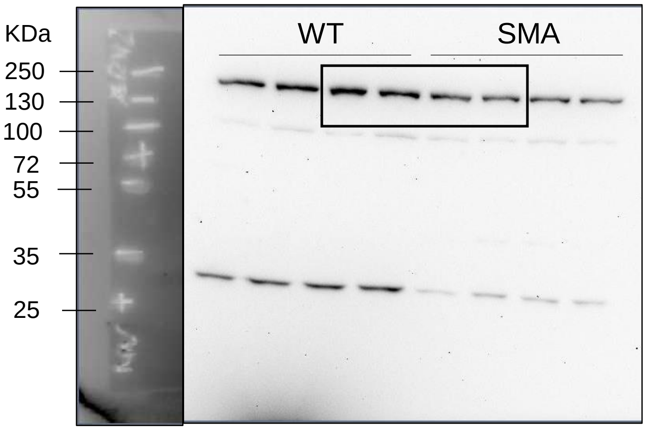

ACTB for DROSHA (Fig 1A)

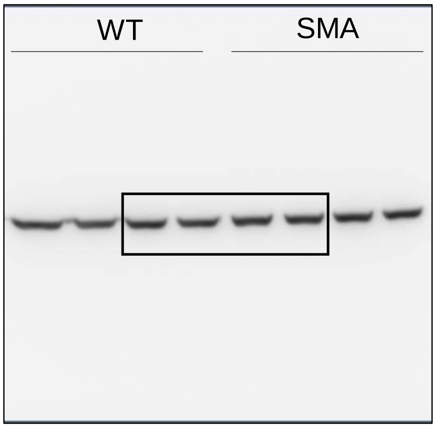

DGCR8 (Fig 1A)

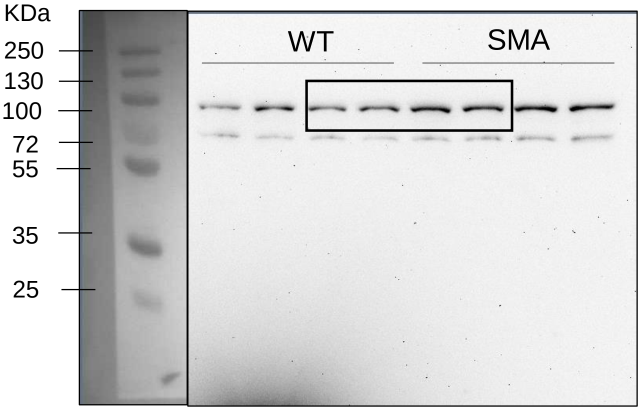

ACTB for DGCR8 (Fig 1A)

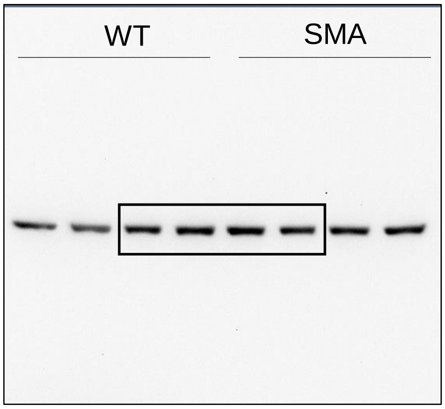

Original Western blot images for Fig. 2

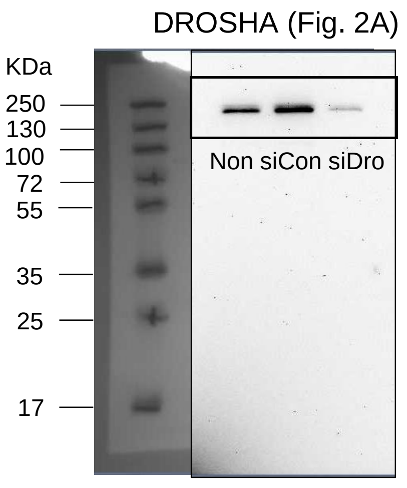

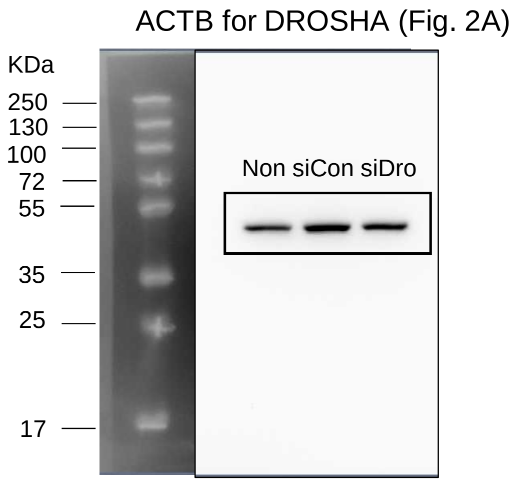

Original Western blot images for Fig 3

DROSHA (Fig 3A)

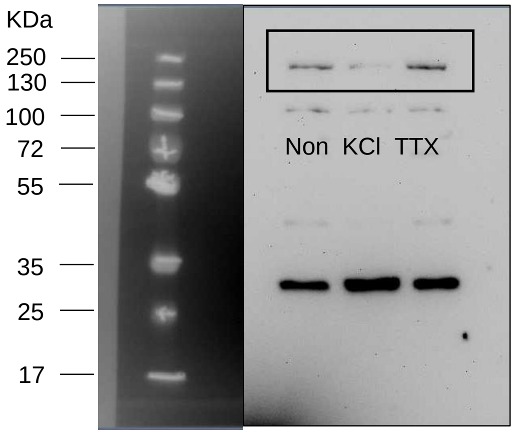

ACTB for DROSHA (Fig 3A)

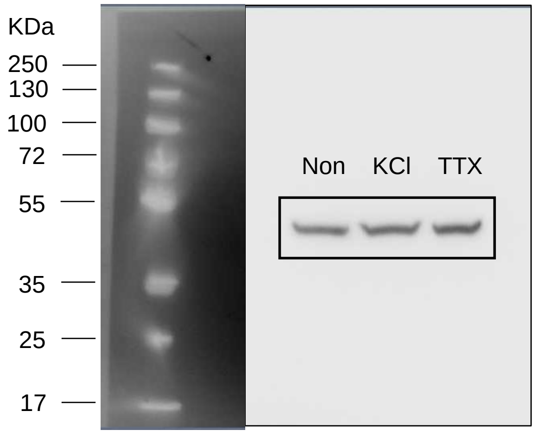

DROSHA (Fig 3B)

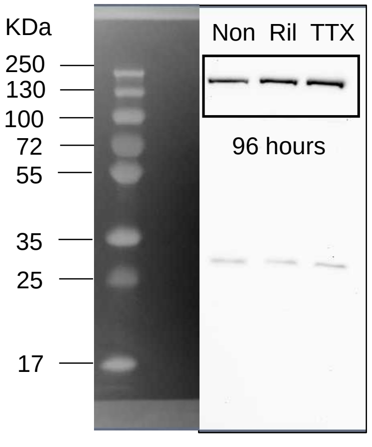

ACTB for DROSHA (Fig 3B)

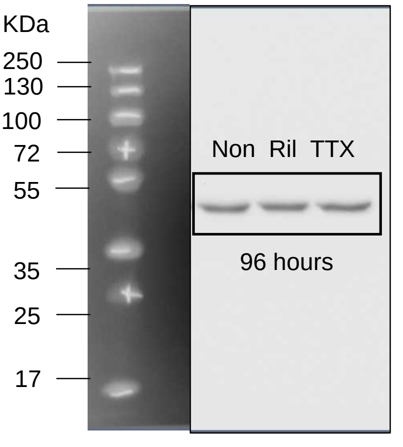

Original Western blot images for Fig 3D

DROSHA (Fig 2D), flipped (left to right, and upside to down)

Order: SMA cyto, SMA nuc, WT cyto, WT nuc, marker

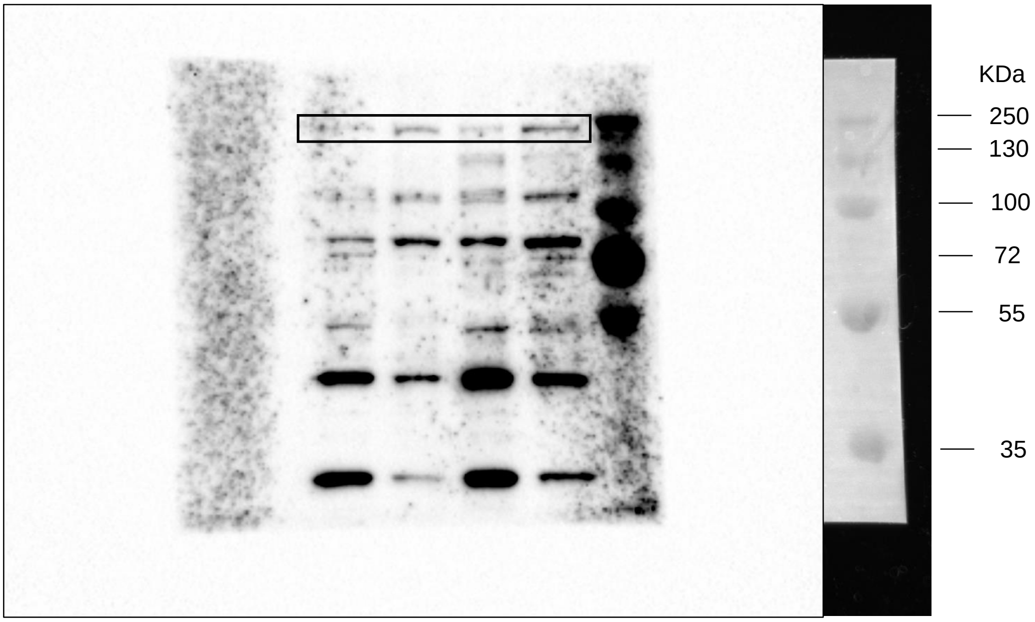

ACTB (Fig 3D), flipped (left to right, and upside to down)

Order: SMA cyto, SMA nuc, WT cyto, WT nuc, marker

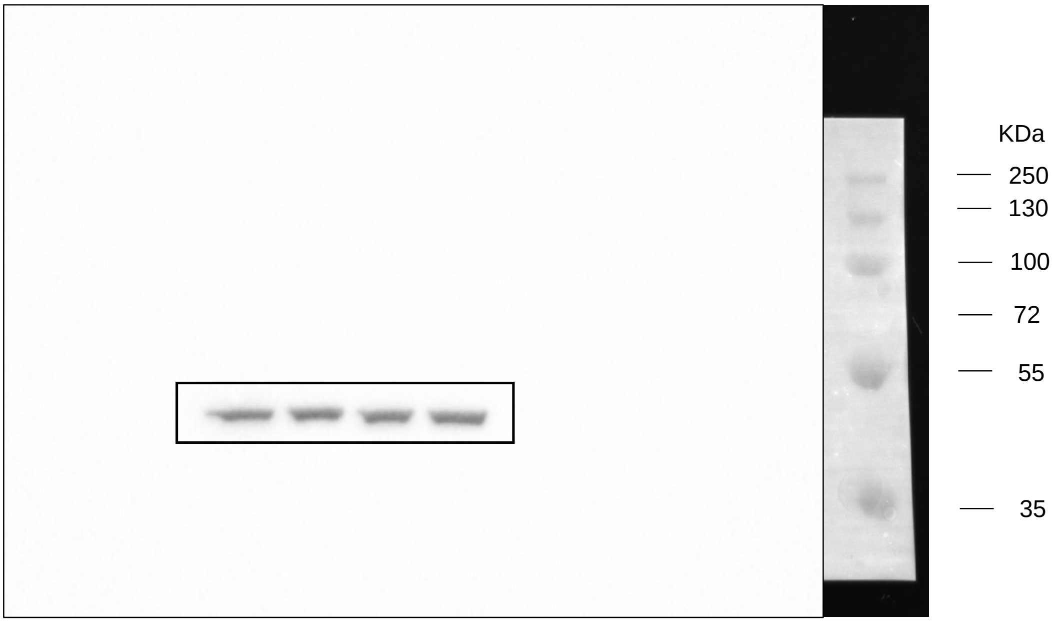

Original Western blot images for Fig 3D

LaminB (Fig 3D), flipped (left to right, and upside to down)

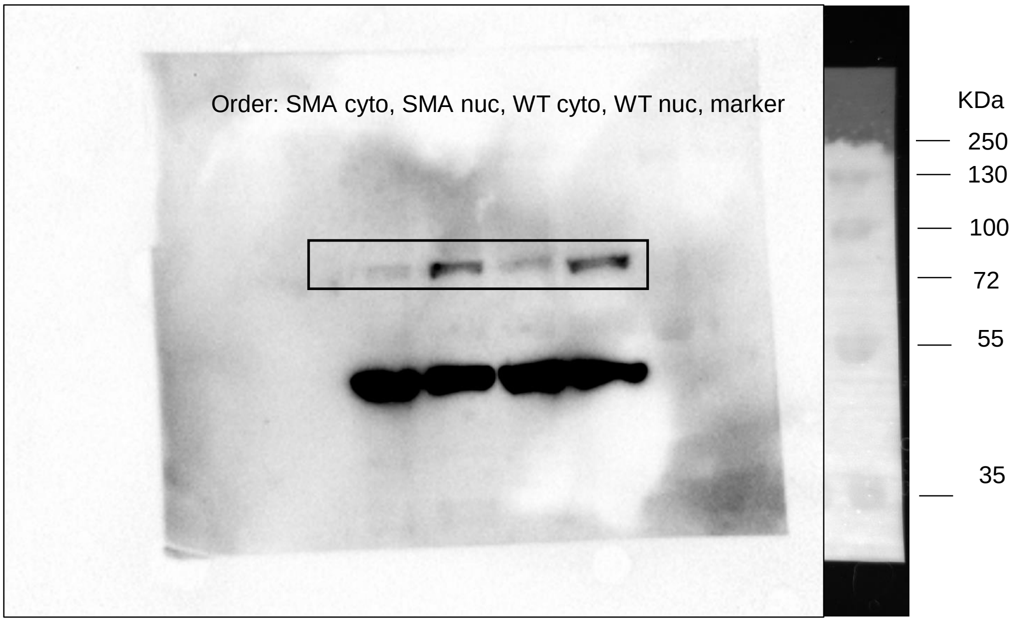

Original Western blot/ gel images for Fig 3E and G

DROSHA (Fig 3E)

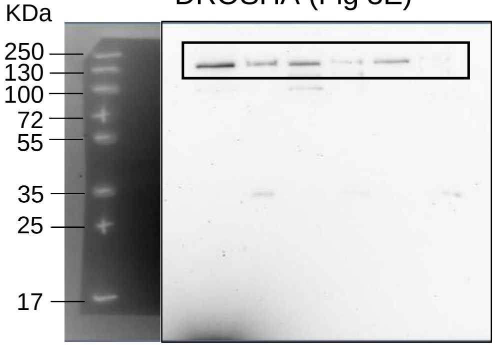

ACTB (Fig 3E)

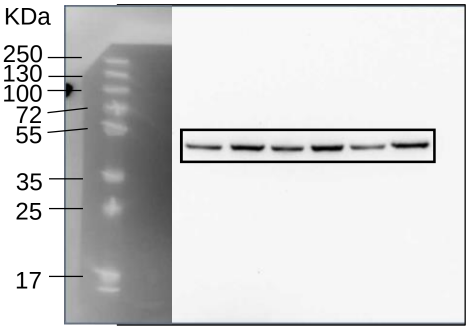

KCl treated, Nuc(con), Cyto (con), Nuc (1h KCl), Cyto (1h KCl), Nuc (24 hrs) and Cyto (24 hrs).

LaminB (Fig 3E)

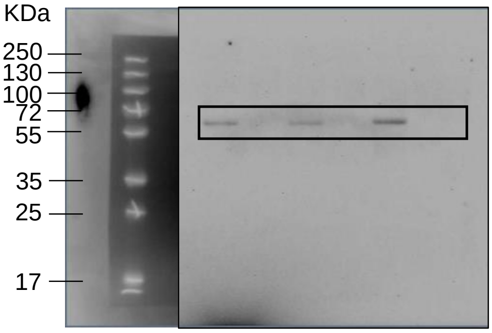

KCl treated, Nuc(con), Cyto (con), Nuc (1h KCl), Cyto (1h KCl), Nuc (24 hrs) and Cyto (24 hrs).

PCR agarose gel picture (Fig 3G)

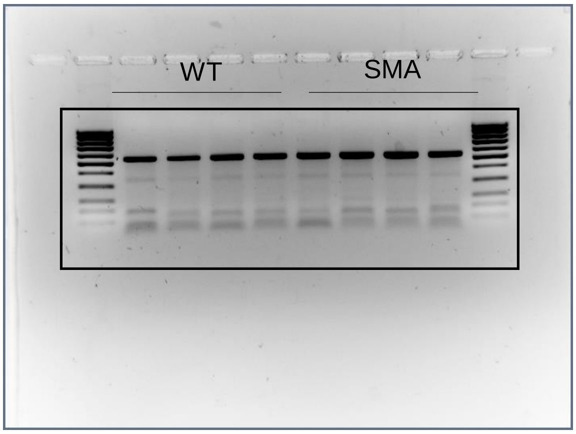

Original Western blot images for Fig 4A

DROSHA (Fig. 4A), flipped (left to right, and upside to down)

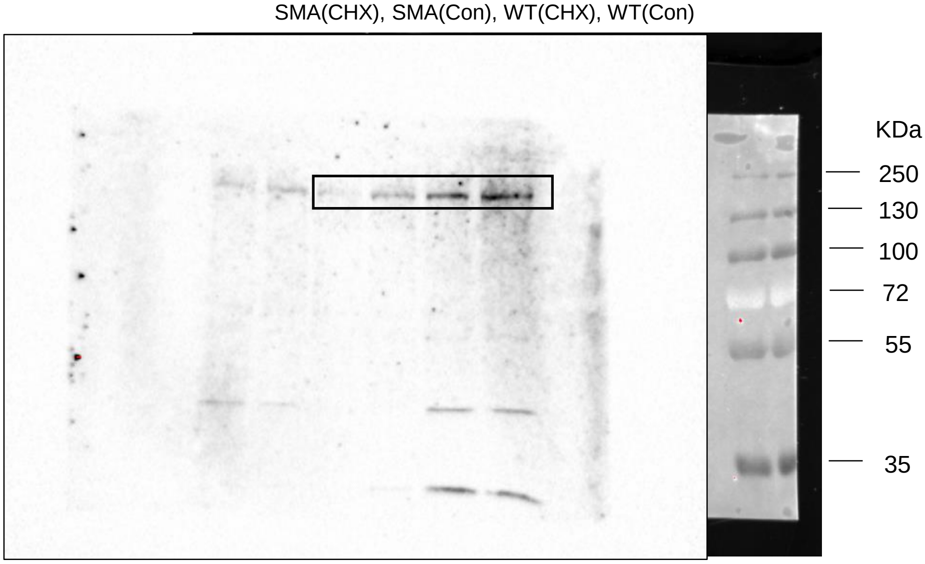

ACTB (Fig. 4A)

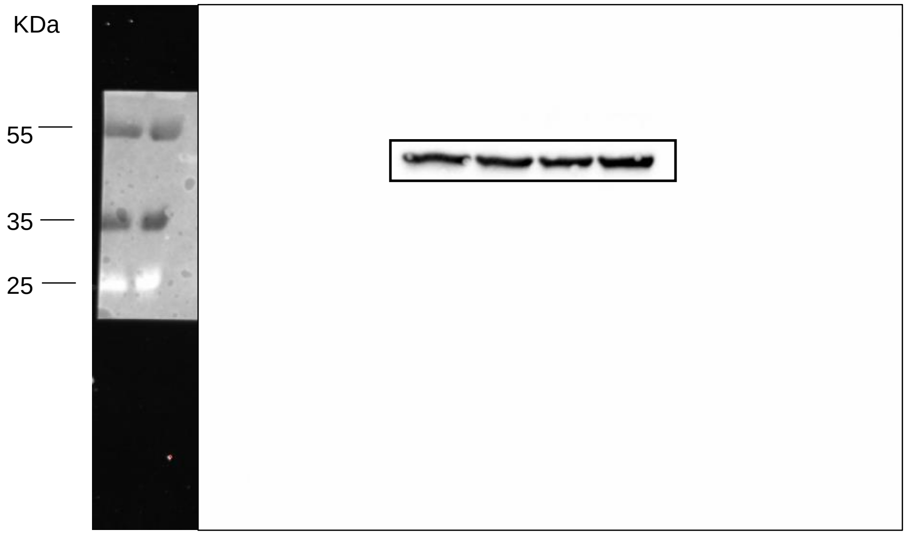

Original Western blot images for Fig 4B

DROSHA (Fig 4B)

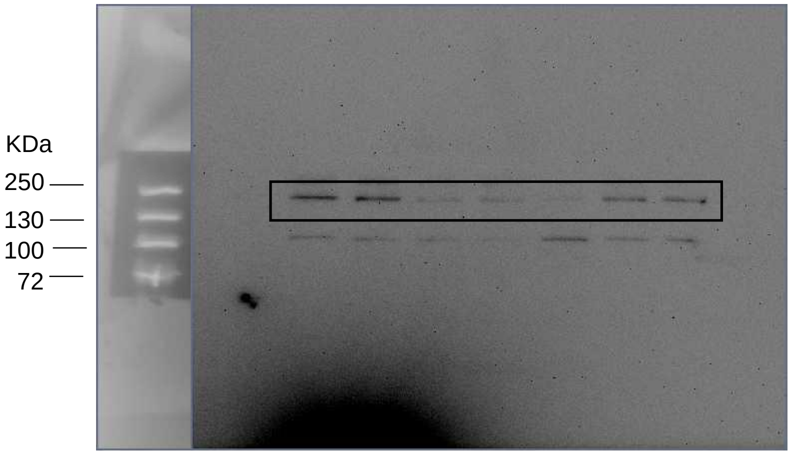

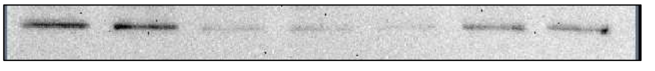

ACTB (Fig 4B)

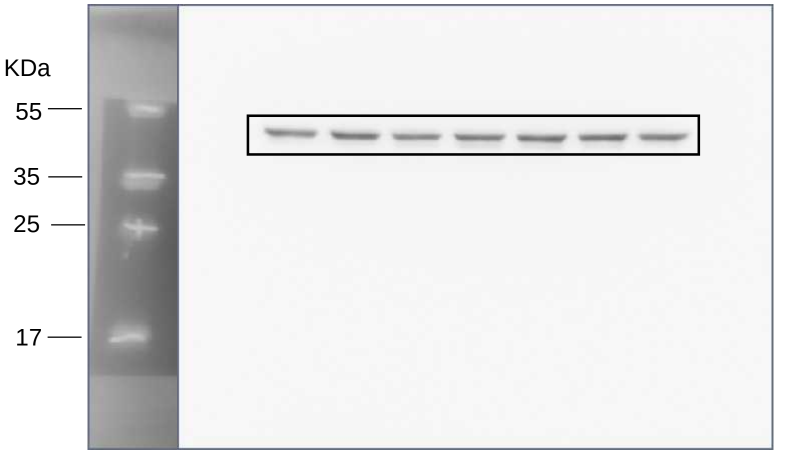

Original Western blot images for Fig. 4C

LC3-I and LC3-II (Fig 4C)

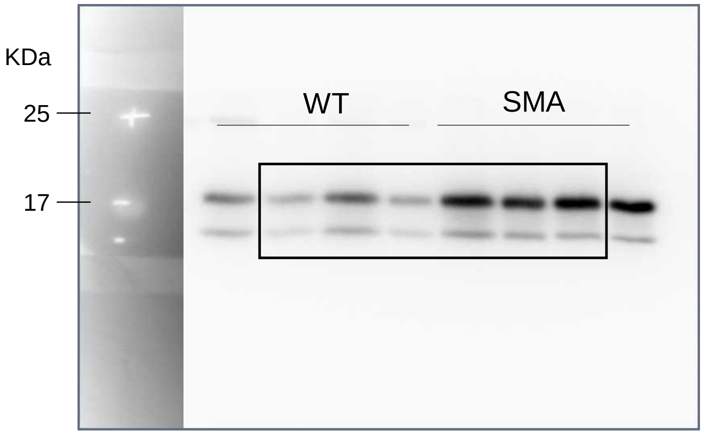

ACTB for LC3-I, -II (Fig 4C)

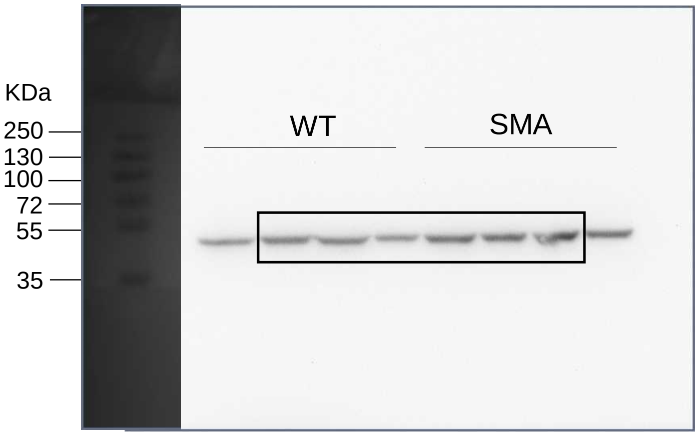

Original Western blot images for Fig. 4C and 4D

P62 (Fig. 4C)

WT SMA

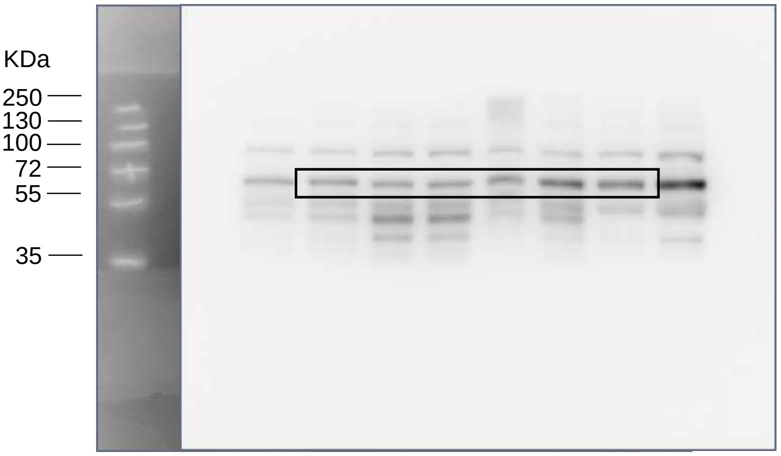

ACTB for P62 (Fig. 4C)

WT SMA

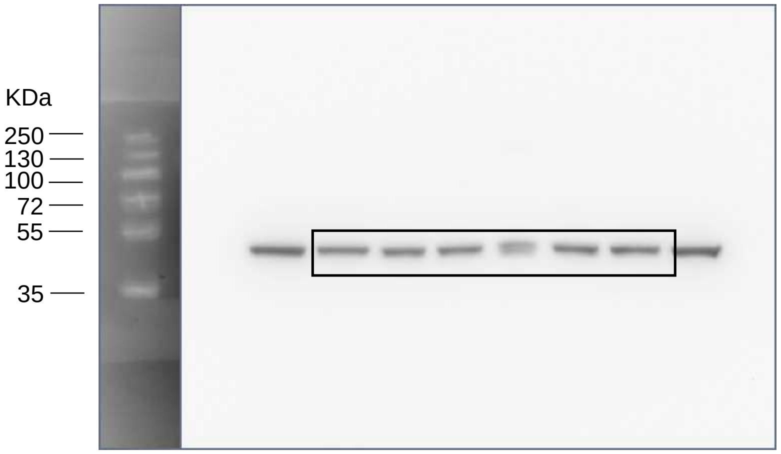

DROSHA (Fig. 4D)

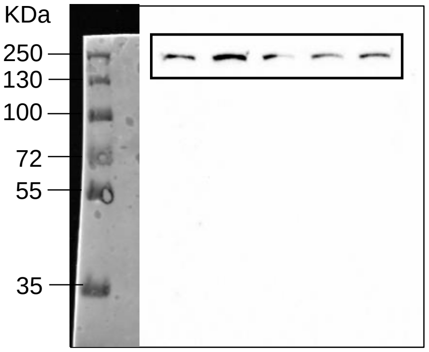

ACTB for DROSHA (Fig. 4D)

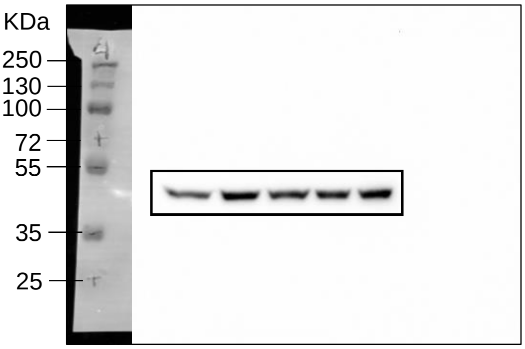

Con, DMSO, Rapamycin, Torin-1 and 10-NCP treated for 24 hours

Original Western blot images for Fig. 4E

DROSHA (Fig. 4E)

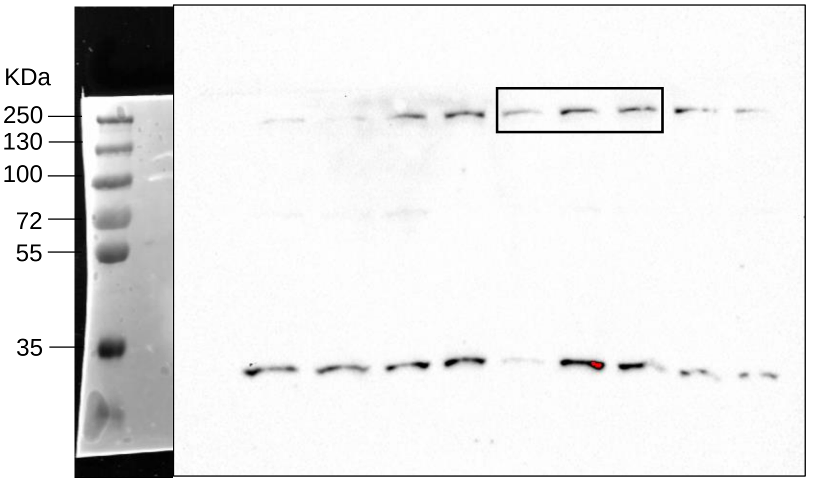

ACTB for DROSHA (Fig. 4E)

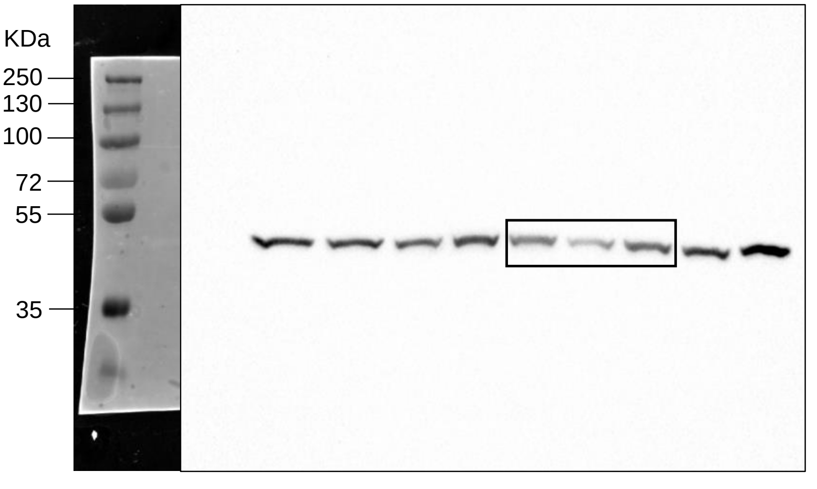

Con, 3-MA and Bafilomycin A treated for 24 hours

Original Western blot images for Fig. 4F and 4G

ATG5 (Fig 4F)

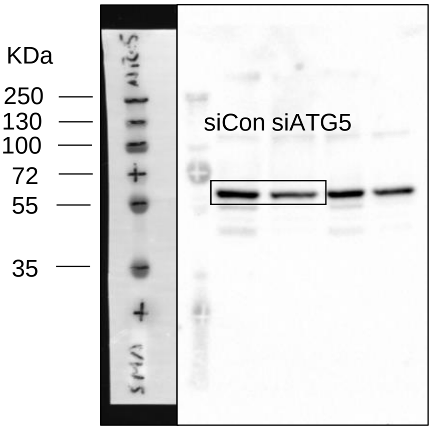

ACTB (Fig 4F)

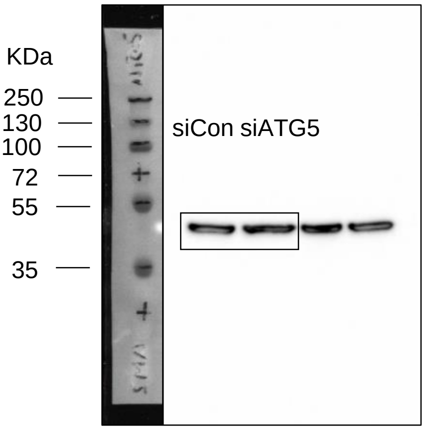

DROSHA (Fig 4G)

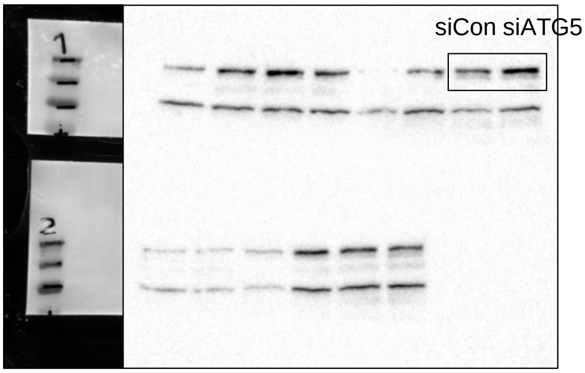

ACTB (Fig 4G)

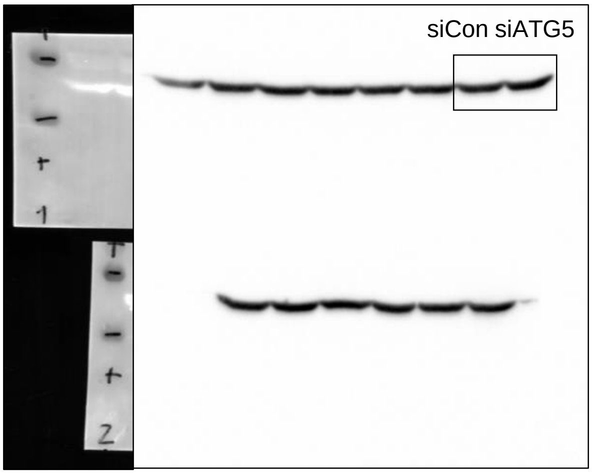

Atg5 knockdown
SMA motor
neurons

Original Western blot images for Fig. 5

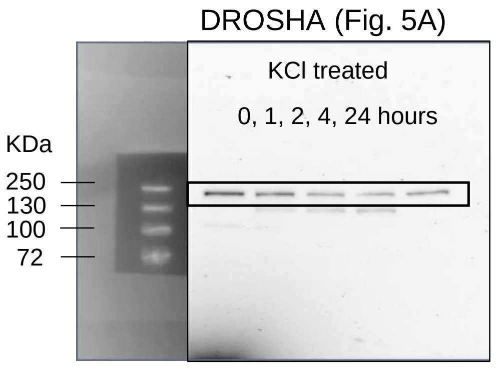

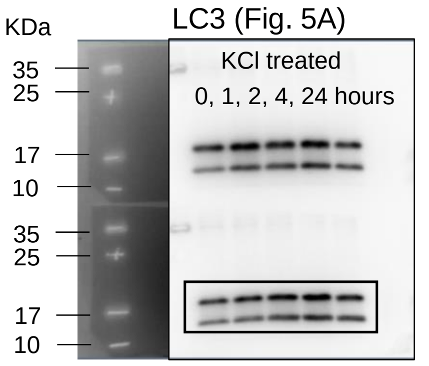

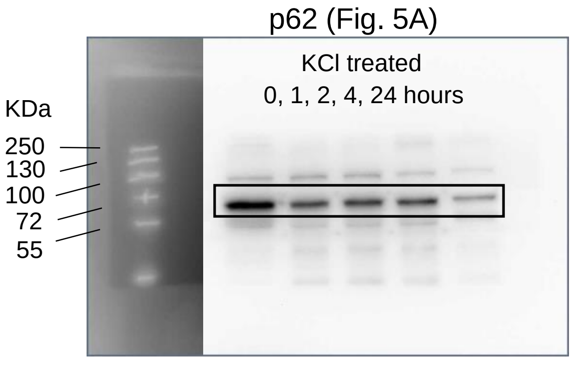

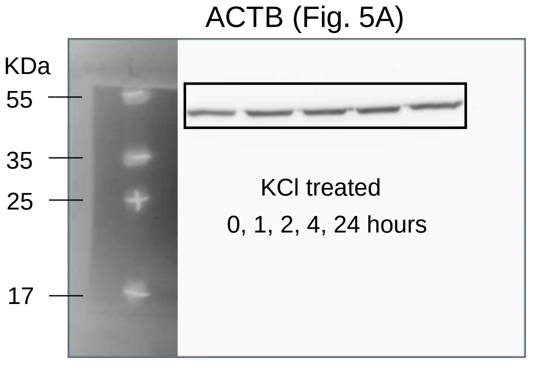

Original Western blot images for Supplementary Fig. 2

AGO2 (Fig S2A)

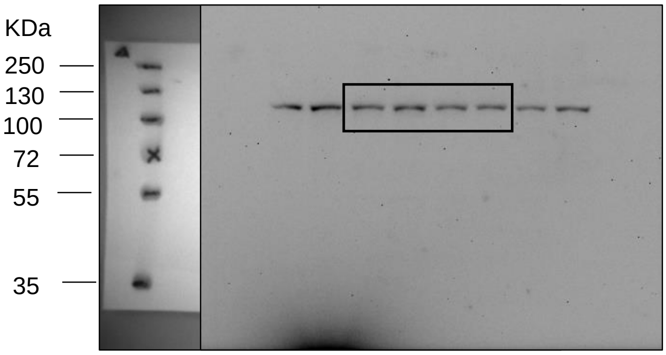

ACTB for AGO2 (Fig S2A)

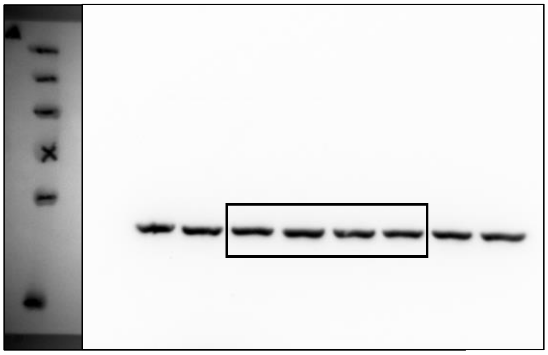

XRN1 (Fig S2A)

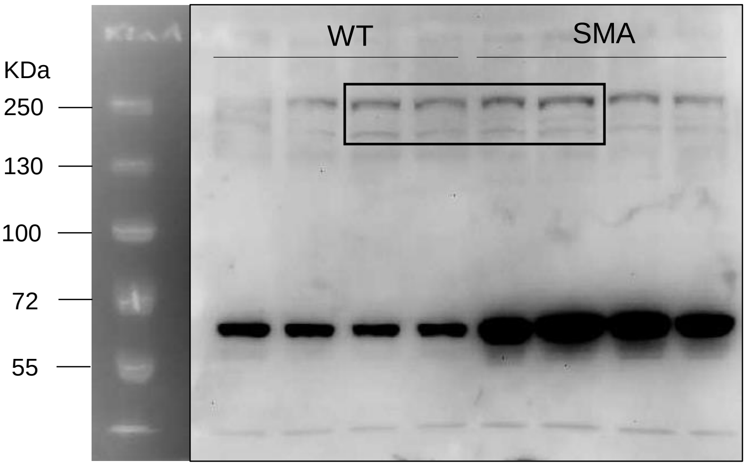

ACTB for XRN1 (Fig S2A)

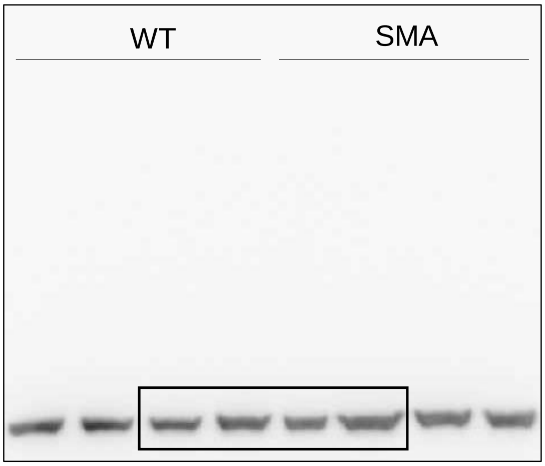

ERI1 (Fig S2A)

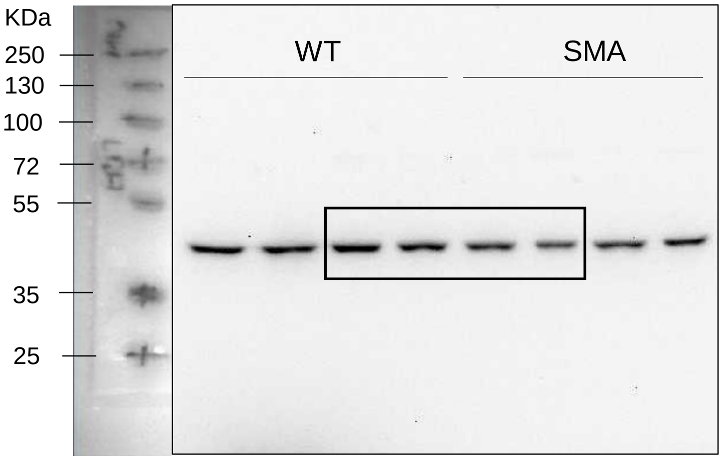

ACTB for ERI1 (Fig S2A)

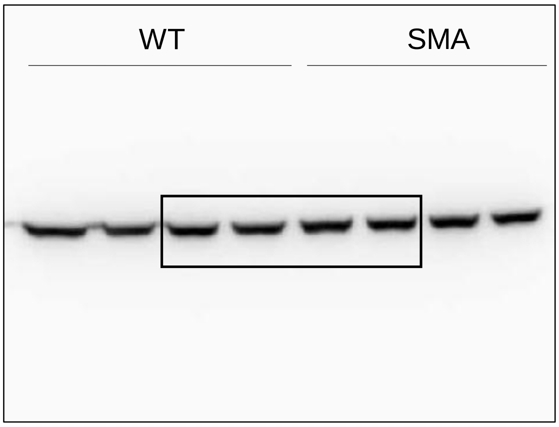

DICER1 (Fig S2A)

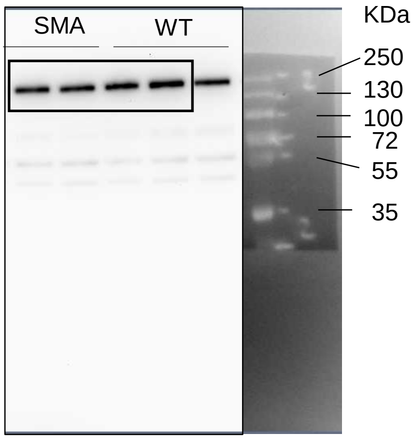

ACTB for DICER1 (Fig S2A)

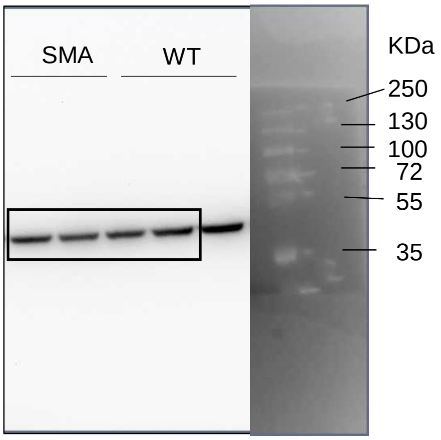

Original Western blot images for Supplementary Fig. 3

DROSHA (Fig. S3A)

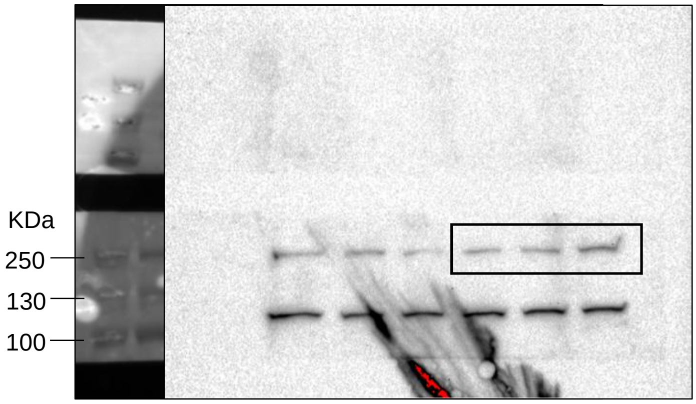

SMN (Fig. S3A)

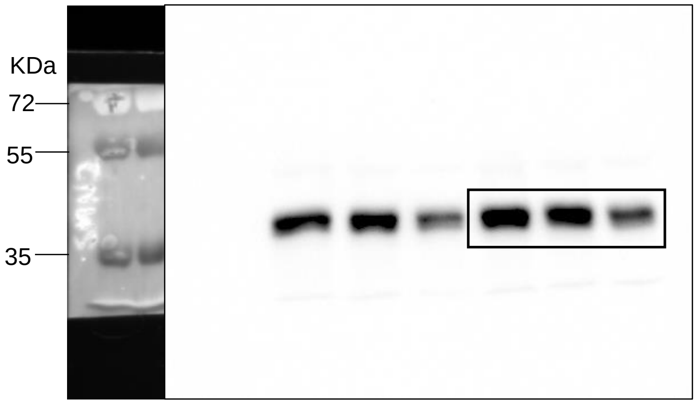

ACTB (Fig. S3A)

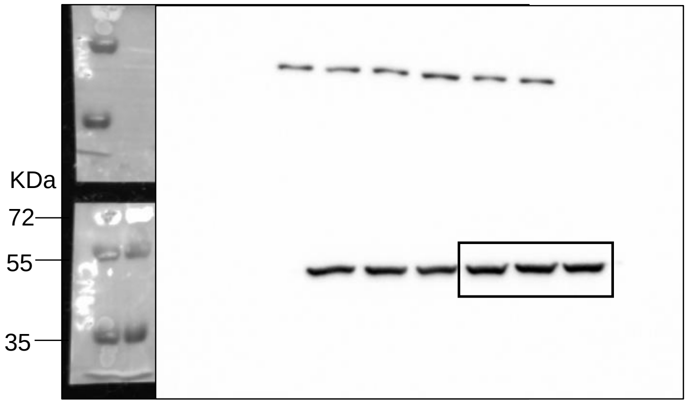

Original Western blot images for Supplementary Fig. 3

SMN (Fig. S3B), image flipped horizontally

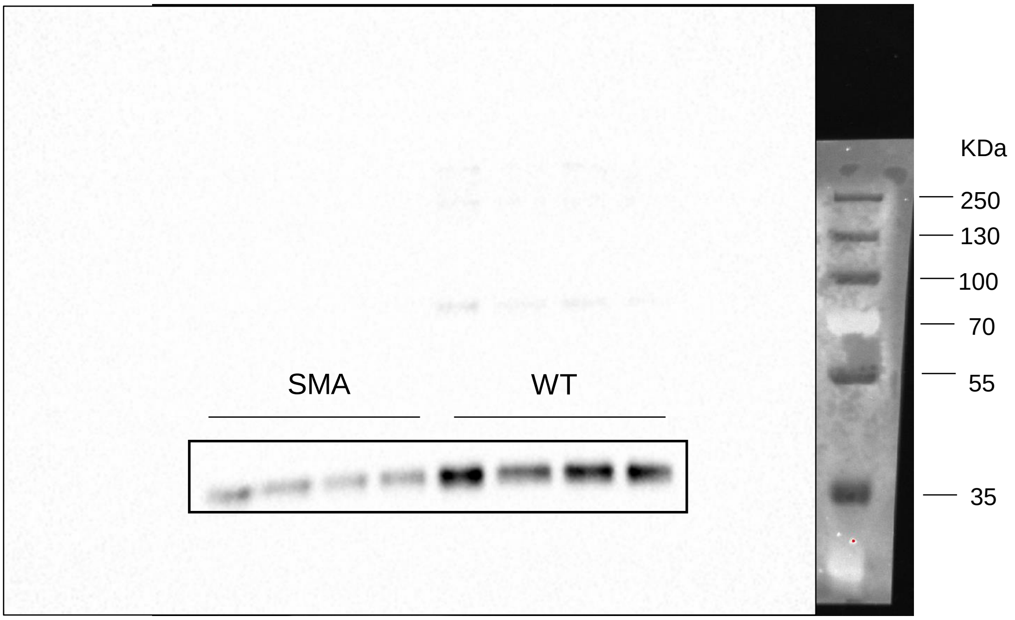

ACTB (Fig. S3B), image flipped horizontally

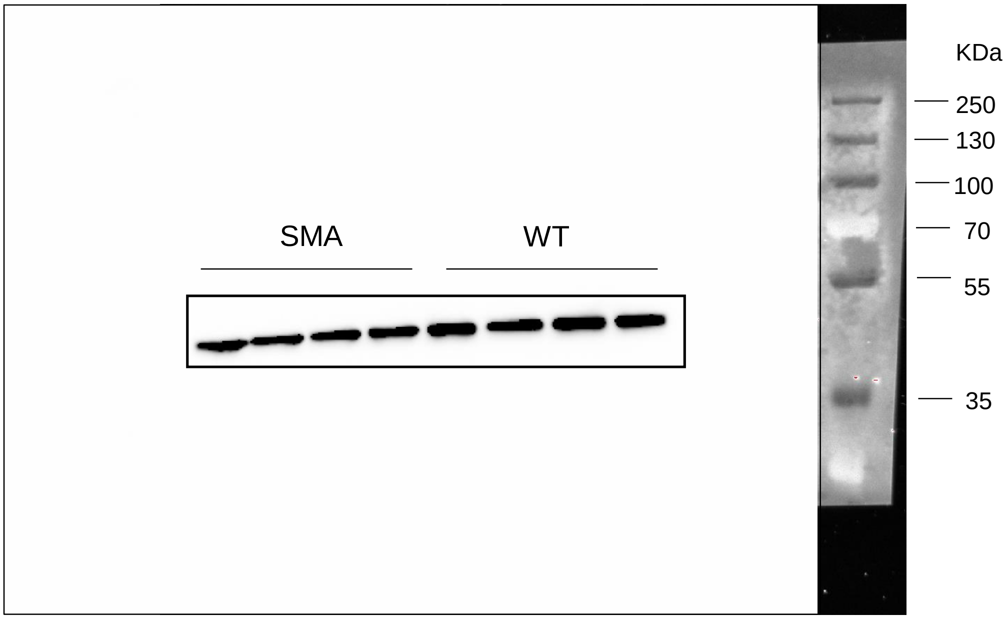

Original Western blot images for Supplementary Fig. 4

DROSHA (Fig. S4A) siRNA testing

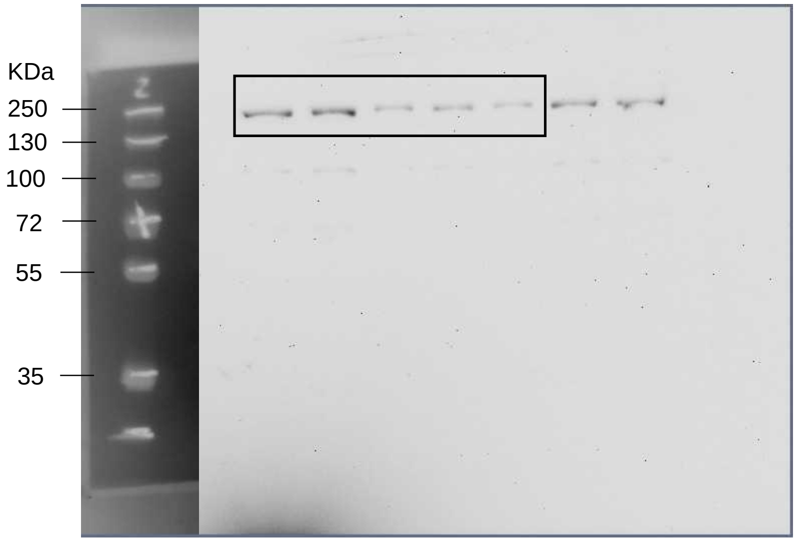

Non treated, control siRNA, siDro1, siDro2 and siDro1 + siDro2

ACTB for DROSHA (Fig. S4A)

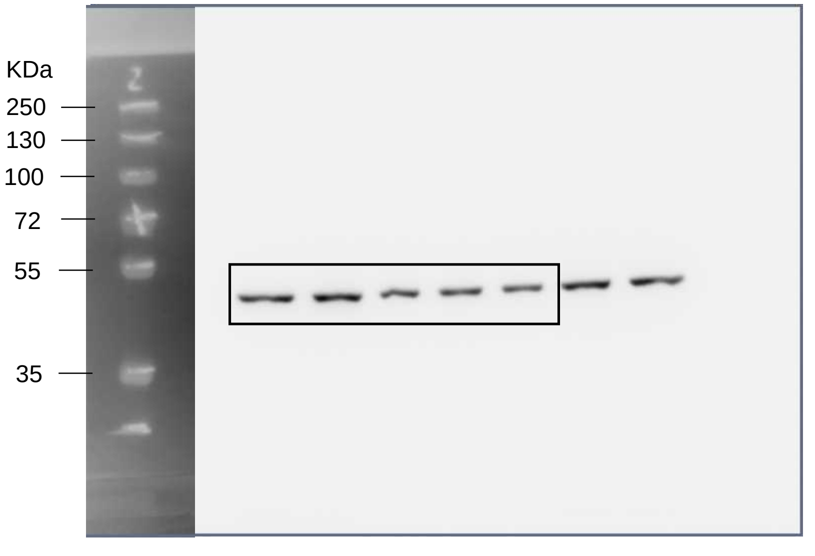

Non treated, control siRNA, siDro1, siDro2 and siDro1 + siDro2

Original Western blot images for Supplementary Fig. 5

DROSHA in primary muscle cells (Fig. S5B)

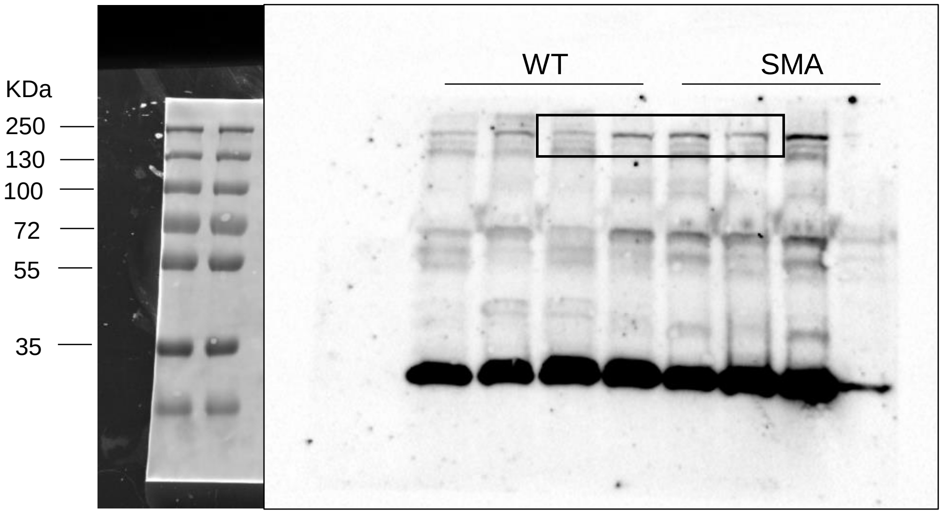

ACTB (Fig. S5B)

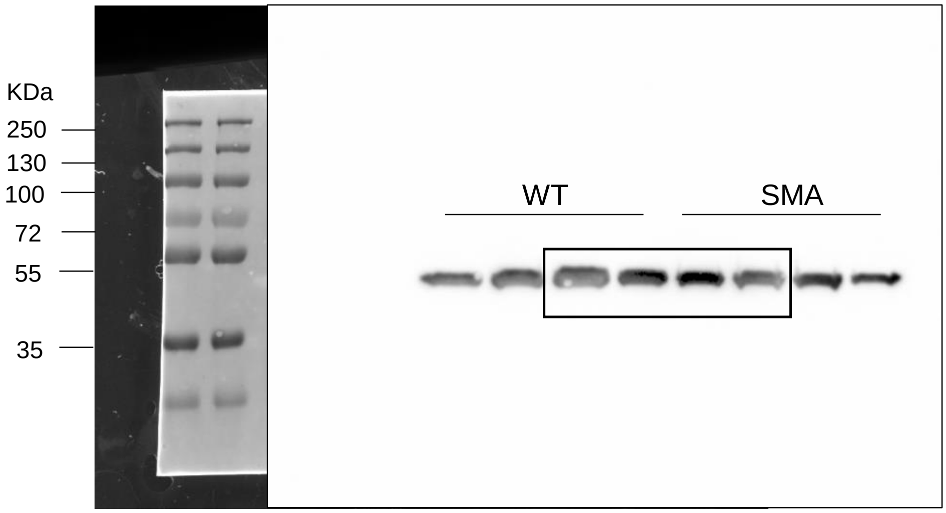

Original Western blot images for Supplementary Fig. 5C

DROSHA in C2C12 cells (Fig. S5C)

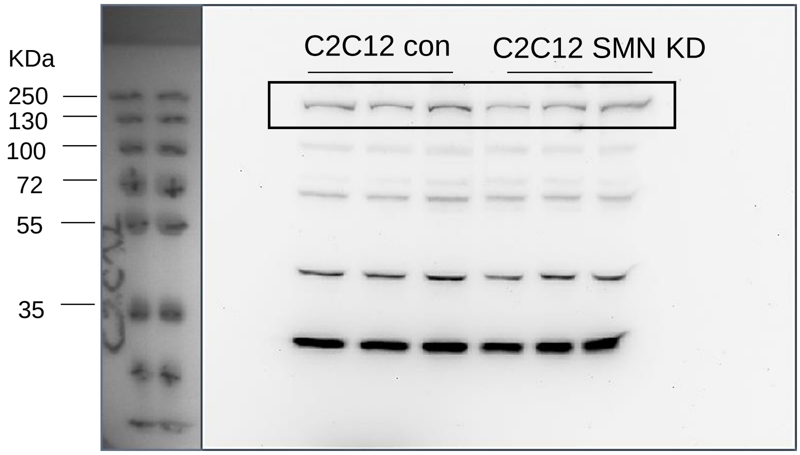

SMN (Same membrane) Fig. S5C

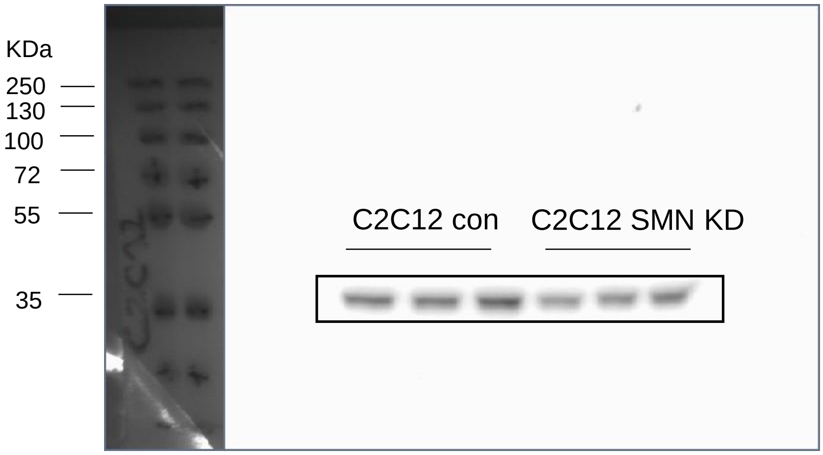

ACTB (same membrane) Fig. S5C

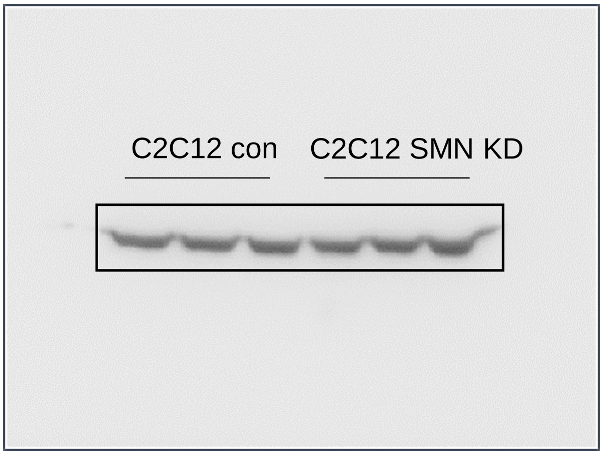

Original Western blot images for Supplementary Fig. 7

DROSHA (Fig. S7)

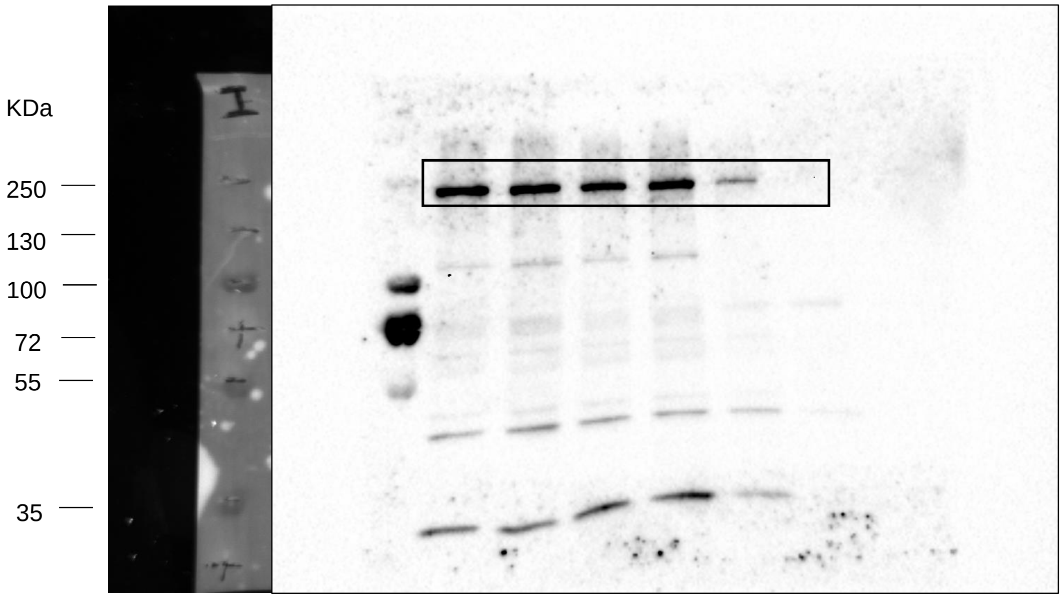

ACTB (Fig. S7)

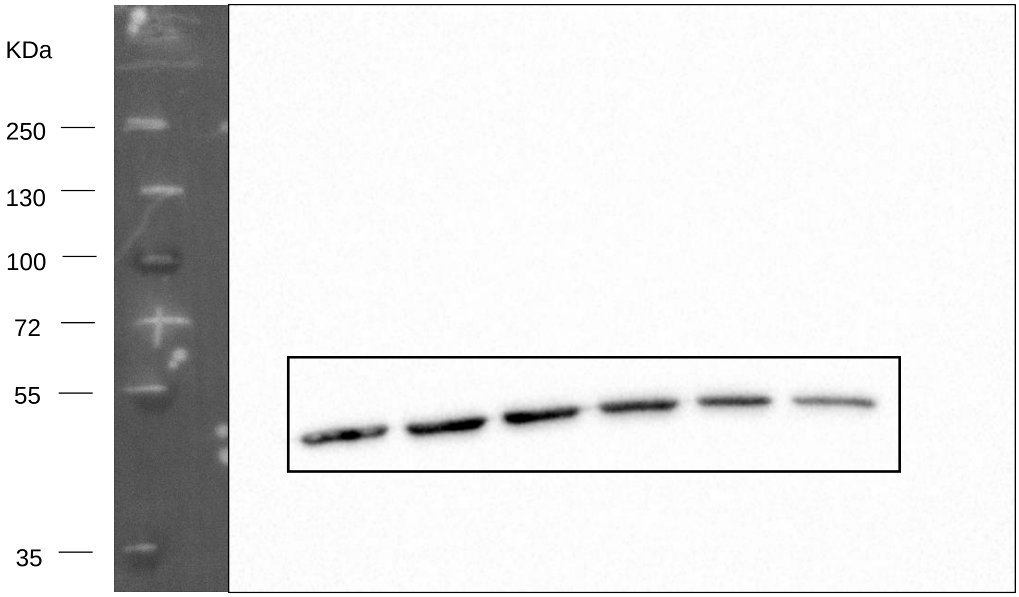

Supplement: Supplementary file 1 — Supplementary information [file 41598_2018_26347_MOESM1_ESM.pdf]
